# Polycomb repressive complex 2 is critical for mouse cortical glutamatergic neuron development

**DOI:** 10.1093/cercor/bhae268

**Published:** 2024-07-03

**Authors:** Laura Currey, Benjamin Mitchell, Majd Al-Khalily, Sarah-Jayne McElnea, Peter Kozulin, Danyon Harkins, Alexandra Pelenyi, Laura Fenlon, Rodrigo Suarez, Nyoman D Kurniawan, Thomas H Burne, Lachlan Harris, Stefan Thor, Michael Piper

**Affiliations:** School of Biomedical Sciences, The University of Queensland, Brisbane, QLD 4072, Australia; School of Biomedical Sciences, The University of Queensland, Brisbane, QLD 4072, Australia; Centre for Advanced Imaging, Australian Institute for Bioengineering and Nanotechnology, The University of Queensland, QLD 4072, Australia; Queensland Brain Institute, The University of Queensland, Brisbane, QLD 4072, Australia; School of Biomedical Sciences, The University of Queensland, Brisbane, QLD 4072, Australia; School of Biomedical Sciences, The University of Queensland, Brisbane, QLD 4072, Australia; School of Biomedical Sciences, The University of Queensland, Brisbane, QLD 4072, Australia; School of Biomedical Sciences, The University of Queensland, Brisbane, QLD 4072, Australia; Queensland Brain Institute, The University of Queensland, Brisbane, QLD 4072, Australia; School of Biomedical Sciences, The University of Queensland, Brisbane, QLD 4072, Australia; Queensland Brain Institute, The University of Queensland, Brisbane, QLD 4072, Australia; Centre for Advanced Imaging, Australian Institute for Bioengineering and Nanotechnology, The University of Queensland, QLD 4072, Australia; Queensland Brain Institute, The University of Queensland, Brisbane, QLD 4072, Australia; Queensland Centre for Mental Health Research, The Park Centre for Mental Health, Wacol, QLD 4076, Australia; School of Biomedical Sciences, The University of Queensland, Brisbane, QLD 4072, Australia; Cancer Neuroscience Laboratory, QIMR Berghofer Medical Research Institute, Brisbane, QLD 4006, Australia; School of Biomedical Sciences, The University of Queensland, Brisbane, QLD 4072, Australia; School of Biomedical Sciences, The University of Queensland, Brisbane, QLD 4072, Australia; Queensland Brain Institute, The University of Queensland, Brisbane, QLD 4072, Australia

**Keywords:** Eed, PRC2, H3K27me3, histone modification, glutamatergic neurons

## Abstract

The Polycomb Repressive Complex 2 (PRC2) regulates corticogenesis, yet the consequences of mutations to this epigenetic modifier in the mature brain are poorly defined. Importantly, PRC2 core genes are haploinsufficient and causative of several human neurodevelopmental disorders. To address the role of PRC2 in mature cortical structure and function, we conditionally deleted the PRC2 gene *Eed* from the developing mouse dorsal telencephalon. Adult homozygotes displayed smaller forebrain structures. Single-nucleus transcriptomics revealed that glutamatergic neurons were particularly affected, exhibiting dysregulated gene expression profiles, accompanied by aberrations in neuronal morphology and connectivity. Remarkably, homozygous mice performed well on challenging cognitive tasks. In contrast, while heterozygous mice did not exhibit clear anatomical or behavioral differences, they displayed dysregulation of neuronal genes and altered neuronal morphology that was strikingly different from homozygous phenotypes. Collectively, these data reveal how alterations to PRC2 function shape the mature brain and reveal a dose-specific role for PRC2 in determining glutamatergic neuron identity.

## Introduction

The cerebral cortex exhibits tremendous cell diversity and elaborate networks of connectivity that are essential for higher-order behavioral and cognitive tasks. Cortical development is driven by the coordinated proliferation and differentiation of neural progenitor cells (NPCs) (Cárdenas and Borrell 2020). This begins with the symmetric proliferative division of neuroepithelial cells within the cortical ventricular zone. As development proceeds, neuroepithelial cells give rise to radial glial cells (RGC) that divide asymmetrically, producing a RGC daughter cell, and either a neuron (direct neurogenesis; early in corticogenesis) or a basal progenitor cell (BP) (indirect neurogenesis; later in corticogenesis) (Cárdenas and Borrell 2020). During late mouse gestation, a subset of RGCs then switches to produce glial cells, such as astrocytes. Precise regulation of the number of neuroepithelial cell and RGC divisions, and of the extent of direct versus indirect neurogenesis, is critical for producing the correct number of neurons and glia in the mature cerebral cortex (Cárdenas and Borrell 2020).

Epigenetic modifiers play a key role in the developing brain by regulating NPC proliferation and differentiation, as well as coordinating postmitotic cellular identity. One epigenetic modifier that plays a critical role in regulating NPC behavior is the Polycomb Repressive Complex 2 (PRC2). PRC2 is responsible for catalyzing mono-, di-, and tri-methylation of lysine 27 (H3K27me1/2/3), primarily on histone H3.3 and to a lesser extent on H3.1 and H3.2 (Banaszynski et al. 2013). H3K27me3 attracts Polycomb Repressive Complex 1, which catalyzes ubiquitylation of lysine 119 of Histone H2A, culminating in transcriptional silencing (Guo et al. 2021). The core components of PRC2 include Enhancer of Zeste Homolog 1/2 (EZH1/2), Suppressor of Zeste 12 (SUZ12), Retinoblastoma binding protein 4/7 (RBBP4/7), and Embryonic ectoderm development (EED). Loss-of-function in any of these core components results in a lack of PRC2 function. Importantly, as PRC2 is the only known complex to catalyze H3K27me1/2/3, it is essential to establish this repressive mark (Margueron and Reinberg 2011), and therefore to mediate pivotal processes including ESC differentiation (Boyer et al. 2006; Lee et al. 2006; Obier et al. 2015), anteroposterior axis specification (Wyngaarden et al. 2011), osteogenesis (Dudakovic et al. 2018), and neurogenesis (Corley and Kroll 2015).

PRC2 function is critical in humans; the gnomAD and EXAC databases show *EED*, *EZH2*, and *SUZ12* to be intolerant to a loss of gene dosage, and hence are haploinsufficient (Lek et al. 2016; Karczewski et al. 2020). Moreover, Weaver, Imagawa-Matsumoto, and Cohen–Gibson syndromes, all of which are overgrowth disorders, are linked to heterozygous, likely neomorphic or hypermorphic gain-of-function mutations to *EZH2*, *SUZ12*, or *EED* (Tatton-Brown et al. 2011; Cohen and Gibson 2016; Imagawa et al. 2018). However, despite the clinical significance of these syndromes, little is known about how PRC2 haploinsufficiency within the nervous system leads to these clinical outcomes.

In mice, constitutive knockout of PRC2 core components causes gastrulation defects and embryonic lethality (Schumacher et al. 1996; Faust et al. 1998; O'Carroll et al. 2001; Pasini et al. 2004; Miao et al. 2020). Conditional knockout of *Eed* in the early CNS using a *Sox1-Cre* driver [expressed in all NPCs from embryonic day (E) 8.5], culminates in embryonic microcephaly arising from premature NPC differentiation (Yaghmaeian et al. 2018; Mora et al. 2022). Likewise, conditional deletion of *Ezh2* from dorsal telencephalic NPCs at E9.5 causes early onset of neurogenesis at the expense of neuroepithelial cell self-renewal, resulting in NPC depletion and consequently fewer late-born neurons (Pereira et al. 2010), a phenotype mirrored by the deletion of *Eed* from dorsal telencephalic NPCs (Telley et al. 2019). Collectively, these studies point to precocious NPC differentiation as a key phenotype arising from abnormal PRC2 function early in CNS development. Numerous other genetic perturbations also cause premature NPC differentiation (Alsiö et al. 2013; Caviness et al. 2003; Hatakeyama et al. 2004; Lange et al. 2009; Najas et al. 2020; Pilaz et al. 2016). However, the adult phenotypes in these mice, including those with abnormal PRC2 function, have received little attention. PRC2 function also contributes to neuronal specification. In the mouse hypothalamus, loss of *Eed* has been reported to have subtle effects on cellular specification, including a reduction in dopamine-, hypocretin-, and Tac2-Pax6-expressing neurons, and an increased number of neurons expressing both glutamatergic and GABAergic markers (Yaghmaeian Salmani et al. 2022). PRC2 is also involved in the survival and function of adult striatal neurons (von Schimmelmann et al. 2016), and in maintaining identity and function of dopaminergic and serotonergic neurons (Toskas et al. 2022). However, whether the loss of *Eed* impacts neuronal specification and identity within the cerebral cortex remains unclear.

Here, we sought to address how precocious NPC differentiation manifests within the adult brain, and whether PRC2 function regulates neuronal identity in the cortex, using mice in which *Eed* had been removed from all cells derived from the dorsal telencephalon (*Emx1-iCre*). Adult homozygous (*Eed-cKO)* but not heterozygous (*Eed-cHet*) knockout mice exhibited microcephaly. Notably, the absence of *Eed* resulted in a reduction of glutamatergic neurons from the dorsal telencephalon, and the aberrant expression of a range of neuronal and nonneuronal genes within these cells, indicative of disrupted cellular identity. In contrast, there were minimal disruptions to gene expression in other cells derived from the dorsal telencephalon, including astrocytes. To our surprise, *Eed-cKO* mice displayed remarkably normal behavior across a range of cortically relevant behavioral tests, some of which were cognitively demanding. Collectively, our findings elucidate the cellular, structural, and transcriptomic deficits arising from PRC2 loss-of-function and identifies heretofore unrealized plasticity with regards to how these mice complete complex behavioral tasks.

## Materials and methods

### Animals

All animals were used with approval from the University of Queensland Animal Ethics Committee (AEC approval number 2022/AE000397). All work was carried out in accordance with the Australian Code of Practice for the Care and Use of Animals for Scientific Purposes and the University of Queensland’s Institutional Biosafety Committee. *Eed*^fl/fl^ mice (Yaghmaeian et al. 2018) were crossed with mice which expressed a codon-improved Cre recombinase gene under control of the *Emx1 promotor* (*Emx1*-iCre positive mice) (Kasherman et al. 2021). This produced pups that were *Eed*^wt/fl^; *Emx1*-iCre positive. *Eed*^wt/fl^; *Emx1*-iCre positive mice were crossed with *Eed*^fl/fl^ mice. This produced pups that were *Eed*^fl/fl^; *Emx1*-iCre positive (*Eed-cKO*, homozygous deletion of *Eed*) and *Eed*^wt/fl^; *Emx1*-iCre positive (*Eed-cHet*, heterozygous deletion of *Eed*). This cross also produced pups that were *Eed*^fl/fl^; *Emx1*-iCre negative and *Eed*^wt/fl^; *Emx1*-iCre negative, which did not have deletion of *Eed* and were used as littermate controls (CTRL). All genotypes were generated at the expected the Mendelian ratios.

### Perfusion

Adult mice were anesthetized by intraperitoneal injection of 0.8 ml of 1:50 Lethabarb (Virbac). A 27-gauge needle was inserted into the left ventricle of the heart, while simultaneously cutting the right atrium and ~15–20 ml of phosphate buffered saline (PBS) was pumped through the body, followed by ~30–40 ml of 4% paraformaldehyde (PFA). The brain was then extracted from the skull and postfixed in 4% PFA for 48 h before storing in PBS at 4 °C.

### Vibratome sectioning

Fixed brains were embedded in 3% noble agar gel and sectioned with the Vibratome VT1000S (Leica) at 50 μm thickness as free-floating sections. Sections were stored in PBS with 0.02% sodium azide at 4 °C.

### Hematoxylin staining

Vibratome-cut sections (50 μm thick) were mounted on SuperFrost Plus microscope slides (Menzel-Glasser, ThermoFisher Scientific) and air dried. Hematoxylin staining was performed using standard protocols as previously described (Kasherman et al. 2021). Imaging was performed using an Aperio SlideScope XT. Images were analyzed with Aperio ImageScope software.

### Immunofluorescent staining

Vibratome-cut sections (50 μm thick) were mounted on SuperFrost Plus slides. Antigen retrieval was performed with a decloaking chamber NxGen (Biocare Medical), at 95 °C for 15 min in citrate buffer. Immunofluorescent staining was performed as described previously (Kasherman et al. 2021); details on the primary and secondary antibodies used are provided in the supplementary methods. Slides were imaged with a Diskovery spinning disc confocal microscope or an Aperio Scanscope XT slidescanner and analyzed with the image J FIJI software.

### Golgi–Cox staining

Freshly perfused brains were incubated in Golgi–Cox solution for 10 d, 30% sucrose for 4 d, then vibratome sectioned (150 μm thick) and mounted onto slides. Once dried, slides underwent development using 0.1 M ammonia, Fujifilm Automatic X-Ray fixer, a series of ethanol dehydrations, and xylene, before being coverslipped. Imaging was performed with a Nikon stereology upright widefield microscope and analysis was performed with the Neurolucida 360 software (version 2019) (Dickstein et al. 2016). Further details on this analysis can be found in the Supplementary Methods.

### Diffusion tensor magnetic resonance imaging (DTMRI)

Mice were perfused as described above; however, the skull was left intact. The samples were immersed in 0.1 M PBS with 0.2% gadobutrol (Gadovist, Bayer) for 8 wk to enhance MRI contrast. Brain data was acquired using a Bruker Ultrashield Plus 700 WB Avance NMR spectrometer. Two sets of scans were obtained: a high-resolution 3D T1 weighted Fast Low Angle Shot for structural information imaging; and 3D Stejskal-Tanner DWI spin-echo in multiple directions for fiber tracking purposes. Several analyses were performed using this data. Volumetric data was investigated in 20 regions using a 3D digital atlas (Ma et al. 2005). Connectome analysis was studied network-wise by graph theory analysis and connection-wise using the Network-Based Statistic Toolbox (NBS) (Zalesky et al. 2010; Wang et al. 2015). DTMRI metrics were measured using the FSL software. White matter fiber density and morphology was investigated with fixel-based analysis using MRTrix3 (Raffelt et al. 2017). Further details on DTMRI tissue preparation, acquisition, preprocessing, and analysis are provided in the Supplementary Methods.

### High resolution episcopic microscopy (HREM)

**Fig. 1:**
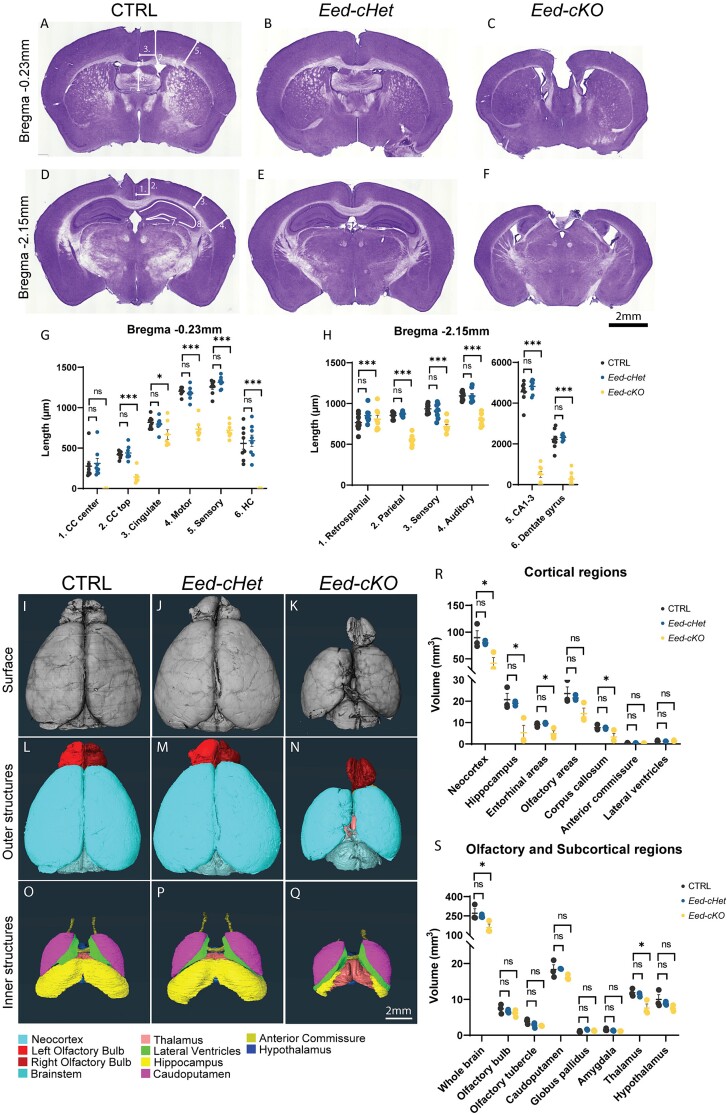
**Loss of *Eed* results in a smaller cortex in the adult**. (A–H) Hematoxylin-stained coronal sections at Bregma −0.23 mm (A–C) and − 2.15 mm (D–F) of control CTRL, *Eed-cHet*, and *Eed-cKO* brains. (G, H) Regions of interest were measured as indicated by annotations on (A, D). CC = corpus callosum, HC = hippocampal commissure. ^*^^*^^*^ = *P* < 0.001, ns = not significant, one-way ANOVA. *n* = 8 CTRL, 8 *Eed-cHet*, 8 *Eed-cKO* (G–H). See also Supplementary Fig. 1 for analysis of additional Bregma sections. (I–S) HREM analysis of CTRL, *Eed-cHet*, and *Eed-cKO* brains. (I–Q) Representative images of HREM 3D reconstruction of the surface (A–C), outer structures (D–F), and inner structures (G–I) of CTRL (A, D, G), *Eed-cHet* (D, E, F), and *Eed-cKO* (G, H, I) brains. (J–K) The volume of major brain regions was measured. ^*^ = *P* < 0.05, ns = not significant, one-way ANOVA. *n* = 3 CTRL, 3 *Eed-cHet*, 3 *Eed-cKO* (R–S).

Fresh brains were fixed in Bouin’s fixative for 24 h then stored in PBS at 4 °C. Processing and sectioning was performed as previously described (Mitchell et al. 2024). Images of the block face were captured following every section. The Amira software package (version 2021.1) was used to compile raw image stacks into whole brain reconstructions, using every fourth image. The software’s “segmentation editor” feature was then used to segment major brain structures (see Fig. 1R–S) and volumetric data for each structure was generated. For the olfactory bulb, only a single olfactory bulb was analyzed as not all brains had both olfactory bulbs intact.

### Single nuclei RNA sequencing (snRNA-seq)

Single nuclei extraction protocol was adapted from (Krishnaswami et al. 2016). Further details are provided in the supplementary methods. Fluorescence activated cell sorting with DAPI was used to filter debris from the nuclei. Sequencing was performed with the 10x Genomics Chromium platform. Analysis was performed with the Seurat toolkit for single cell genomics on R Studio (Hao et al. 2021). Additionally, Azimuth (Hao et al. 2021) was used to transfer annotations from CTRL to *Eed-cKO* glutamatergic neurons and gene ontology (GO) analysis was performed with the clusterProlifer function enrichGO (Wu et al. 2021). Further details on snRNA-seq analysis are provided in the Supplementary Methods.

### Real-time polymerase chain reaction

Mice were euthanized with cervical dislocation and the cortex was immediately dissected and snap-frozen in liquid nitrogen, then stored at −80 °C. RNA was extracted with the RNeasy Mini Kit (Qiagen). The cDNA library was prepared with the QuantiTect Reverse Transcription kit (Qiagen). The PCR reaction was prepared with the QuantiNova SYBR Green PCR kit (Qiagen) and run on the QuantStudio Real-Time PCR system.

### Behavior

All mice were group-housed (2–5 mice per cage) within individually ventilated cages under a 12-h light/dark cycle (lights on 0700 h) in a room maintained at constant temperature (21 °C) and humidity (60%) within a PC2 animal facility. Three different cohorts of mice underwent behavior analysis. The first and second cohort both consisted of 4 females and 4 males per genotype, amounting to a total of 8 CTRL, 8 *Eed-cHet*, and 8 *Eed-cKO* mice. The third cohort consisted of 4 females and 5 males per genotype, amounting to a total of 9 CTRL, 9 *Eed-cHet*, and 9 *Eed-cKO* mice. The first cohort underwent SHIRPA and the 3-chambered social interaction test. The second cohort underwent the activity monitor, light/dark box, elevated plus maze, and active place avoidance tests. Finally, the third cohort underwent the cognitive flexibility test. Details for each behavior test is included in the Supplementary Methods.

## Results

### Loss of *Eed* results in a smaller adult brain

Conditional ablation of PRC2 genes from CNS progenitor cells has been shown to cause severe reduction of cortical size in the embryo (Pereira et al. 2010; Yaghmaeian et al. 2018; Telley et al. 2019). However, how does this phenotype manifest in the adult brain? To investigate this question we employed an *Eed^fl/fl^;Emx1*-iCre model, in which *Eed* was conditionally removed from all dorsal telencephalic NPC-derived cells including glutamatergic neurons, astrocytes, and most cortical oligodendrocytes, but not from cell types that arise from other regions during development, such as interneurons, microglia, and a proportion of oligodendrocytes (Gorski et al. 2002; Kessaris et al. 2006). Consistent with previous embryonic data, we found that *Eed-cKO* brains had major reductions in tissue size throughout cortical and hippocampal regions compared to controls (Fig. 1, Supplementary Fig. 1). The width of the *Eed-cKO* cortical plate was reduced in the cingulate, motor, sensory, auditory, and visual cortices (Fig. 1G, H). The retrosplenial cortex was also reduced in more caudal sections (Supplementary Fig. 1Q). By contrast, there was no significant difference between control and *Eed-cHet* brains.

The dramatic reduction in size of the dorsal telencephalon of cKO mice prompted us to examine if the 3 major forebrain commissures were intact, given that they arise, at least in part, from tissue derived from Emx1-expressing progenitor cells. The corpus callosum of *Eed-cKO* mice was severely disrupted, and callosal axons were rarely observed to cross the midline (Fig. 1C). The only region we observed *Eed-cKO* callosal axons decussating was at approximately Bregma 0.85 mm, at which point the dorsoventral height of the corpus callosum at the midline measured just 7% of the average control. The hippocampal commissure was difficult to identify in *Eed-cKO* hematoxylin-stained sections (Fig. 1C). This was likely due to the hippocampal phenotype, with the dentate gyrus and the CA1–3 regions either severely reduced or unidentifiable (Fig. 1F). The penetrance was 100%, with all animals analyzed showing cortical defects. By contrast, expressivity was variable, particularly in the hippocampus where phenotypes ranged from a complete absence of hippocampal structures to severely diminished, but present, CA or dentate gyrus regions (Supplementary Fig. 2E–T). There was no clear correlation between sex and severity of phenotype. In contrast to the corpus callosum and hippocampal commissure, the anterior commissure was not significantly altered in either heterozygous or homozygous animals (Supplementary Fig. 1O).

We next investigated how the reduction in cortical size manifested at a more global level with HREM, a technique that enables 3D volume rendering to be performed upon embedded and sectioned tissues (Fig. 1I-S). Consistent with our hematoxylin analyses, *Eed-cKO* brains were severely reduced in volume. On average, the whole brain volume (excluding the olfactory bulbs and cerebellum) was 43% smaller in *Eed-cKO* brains compared to controls. In line with the hematoxylin results, the volume of the neocortex, hippocampus, and hippocampal commissure (including fornix and fimbria) was reduced in *Eed-cKO* mice. Although *Emx1* is expressed during the development of the olfactory bulb and in a proportion of olfactory cells in the adult (Briata et al. 1996; Kohwi et al. 2007), there was no significant reduction in the volume of the olfactory bulb, olfactory tubercle, or olfactory cortex of *Eed-cKO* mice. The thalamus was also smaller in *Eed-cKO* mice, likely an indirect effect reflective of the numerous reciprocal connections between the cortex and thalamus, since the thalamus retains *Eed* expression in this model. We did not detect any differences between control and *Eed-cHet* brains, neither with regards to whole brain volume, nor discrete regions of the telencephalon or diencephalon. Volumetric magnetic resonance imaging further confirmed the reduction in a variety of cortical and subcortical structures of adult *Eed-cKO* mice (Supplementary Fig. 2A–D). Collectively these findings indicate that removal of *Eed* from NPCs of the developing dorsal telencephalon culminates in a dramatic reduction in the size of the adult brain.

### Loss of *Eed* causes deficits in the cortical plate

**Fig. 2:**
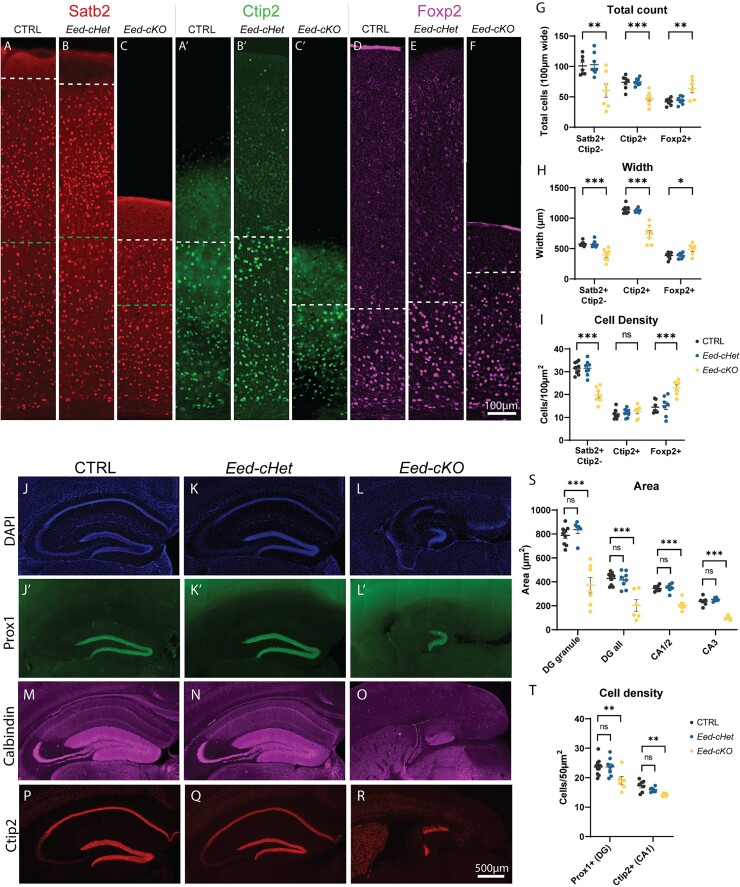
**Loss of *Eed* causes deficits in cortical lamination and hippocampal morphology.** (A–F) Immunofluorescence staining of Satb2 (A–C), Ctip2 (A’-C′), and Foxp2 (D–F) in the somatosensory cortex of adult CTRL, *Eed-cHet*, and *Eed-cKO* mice. Satb2 and Ctip2 were analyzed on the same brain sections, whereas Foxp2 was stained from separate sections. Dashed lines represent the border between positive and negative layers for each marker. (G) The total number of positive cells within a 100 μm wide region spanning all layers of the cortical plate was counted. (H) The width of Ctip2^+^, Ctip2-/Satb2^+^, and Satb2^+^ cortical layers was measured. (I) Cell density was obtained by counting number of cells within a 100 μm^2^ region within layer 6 (Foxp2), layer 4/5 (Ctip2), or layer 2/3 (Satb2). ^*^^*^^*^ = *P* < 0.001, ^*^^*^ = *P* < 0.01, ^*^ = *P* < 0.05, ns = not significant, one-way ANOVA. *n* = 7 CTRL, 7 *Eed-cHet*, 7 *Eed-cKO* (G–I). (J–R) Immunofluorescence staining of DAPI (J–L), Prox1 (J′–L′), calbindin (M–O), and Ctip2 (P–R) in the hippocampus of CTRL, *Eed-cHet*, and *Eed-cKO* adult mice. DAPI and Prox1 were co-stained, whereas calbindin and Ctip2 were stained from separate sections. (S) The area of the dentate gyrus granule cell layer (“DG granule,” indicated by Prox1^+^ staining), dentate gyrus region (“DG all,” granule, molecular, and polymorph layers combined, as indicated by calbindin^+^ staining in the dentate gyrus region), CA1/2 pyramidal layer (Ctip2^+^) and CA3 pyramidal layer (Ctip2^−^, DAPI^+^) were measured. (T) Cell density of Prox1^+^ cells in the dentate gyrus and Ctip2^+^ cells in the CA1 were measured. ^*^^*^^*^ = *P* < 0.001, ^*^^*^ = *P* < 0.01, ns = not significant, one-way ANOVA. *n* = 10 CTRL, 8 *Eed-cHet*, 8 *Eed-cKO* (Prox1 area measurements); *n* = 10 CTRL, 8 *Eed-cHet*, 6 *Eed-cKO* (Prox1 cell count). *n* = 9 CTRL, 6 *Eed-cHet*, 6 *Eed-cKO* (calbindin). *n* = 7 CTRL, 6 *Eed-cHet*, 6 *Eed-cKO* (Ctip2, DAPI).

Premature differentiation of NPCs is known to contribute to microcephaly due to the early depletion of the proliferative pool that results in a dearth of later-born cortical neurons in particular (Fenlon 2022). To investigate lamination in our model, we performed immunofluorescent staining of the neuronal markers Satb2, Ctip2, and Foxp2 (Fig. 2A–I). Satb2 is expressed in cortical layers 2–6, Ctip2 is expressed in layers 5/6, and Foxp2 is expressed in layer 6 and a small population of layer 5 neurons, allowing different cortical layers to be distinguished (Ferland et al. 2003; Molyneaux et al. 2007; Leone et al. 2015). Consistent with the hematoxylin stains, the *Eed-cKO* mice had a thinner cortical plate, with decreased width in both Satb2^+^/Ctip2^−^ and Ctip2^+^ layers, denoting upper and mid-lower layers, respectively. In line with this, *Eed-cKO* mice also had fewer Satb2^+^/Ctip2^−^ and Ctip2^+^ cells compared to controls (Fig. 2G). The decrease in laminar neurons was most pronounced in the later-born Satb2^+^/Ctip2^−^ layer. Remarkably however, although we observed cells expressing Foxp2 in the lower part of the cortical plate of the mutant, which likely represents layer 6, we also observed cells expressing this factor sporadically across the cortical plate. As such, the width of the Foxp2^+^ cell layer was thicker in *Eed-cKO* mice compared to controls, and there were more Foxp2^+^ cells in total, despite the overall thinner cortical plate (Fig. 2H). Additionally, the cell density of Foxp2^+^ cells was also increased in *Eed-cKO* mice, whereas cell density of Satb2^+^/Ctip2^−^ cells in layer 2–3 was decreased, and Ctip2^+^ cells in layer 4–5 was unchanged. There were no significant differences in *Eed-cHet* mice compared to controls. We also observed that the delineation between cortical laminae was abnormal in *Eed-cKO* mice. For instance, the boundary between Foxp2, Ctip2, of Satb2 positive and negative layers formed a distinct, straight line in *Eed-cHet* and control mice. However, in *Eed-cKO* mice, this boundary was erratic and often ambiguous (Supplementary Fig. 3A–I).

Next, we investigated astrocytes and oligodendrocytes via GFAP and Olig2 expression, respectively (Supplementary Fig. 3J–W). We observed an increase in GFAP expression in the motor/cingulate cortex of *Eed-cKO* mice, but not in other cortical regions. In contrast, the density of Olig2^+^ cells was decreased in *Eed-cKO* mice, suggesting that the number of oligodendrocytes may be reduced. In contrast to *Eed-cKO* mice, there was no difference in GFAP or Olig2 staining in *Eed-cHet* mice compared to control. Interneurons were investigated with calbindin immunofluorescent stains, which labels a subset of interneurons (Supplementary Fig. 3X–AD). The density of calbindin^+^ cells was increased in *Eed-cKO* mice. As interneurons are not affected by embryonic *Eed* deletion in this model, this increased cell density is likely due to a normal number of interneurons migrating into a smaller cortex. Finally, we investigated blood vessel integrity via PDGFRA immunostaining, which labels vascular endothelial cells as well as oligodendrocyte precursor cells (OPCs), and cell death via cleaved caspase 3 expression. No abnormalities in blood vasculature or cell death were observed (Supplementary Fig. 3 AE–AM). Collectively, these data reveal 2 key phenotypes within the adult *Eed-cKO* brain. Firstly, the reductions in cortical plate width and glutamatergic neuron and oligodendrocyte numbers of the mutant are consistent with the previously reported embryonic precocious neurogenic and microcephalic phenotypes (Yaghmaeian et al. 2018; Mora et al. 2022). Secondly, although lamination appears grossly normal in the *Eed-cKO* brain, the expression of Foxp2 more broadly within the cortical plate is suggestive of potential abnormal glutamatergic cellular identity in cortical neurons as a result of deficient H3K27 methylation within the developing brain.

### Loss of *Eed* causes altered hippocampal morphology

The dramatic hippocampal phenotype observed at a gross morphological level (Fig. 1) led us to next investigate the hippocampus in more detail. To this end, 3 hippocampal markers were used: Prox1 to stain the granule cell layer of the dentate gyrus, calbindin to stain the molecular, granule, and polymorphic layers of the dentate gyrus, and Ctip2 to stain the CA1/2 regions (Fig. 2J–T). The CA3 region was inferred by DAPI^+^, Ctip2^−^ staining. *Eed-cKO* sections were highly abnormal, often displaying a complete absence of Prox1^+^ cells in the presumptive dentate gyrus. Of the 10 *Eed-cKO* biological replicates examined, 3 samples did not exbibit any Prox1^+^ cells, 4 samples only had Prox1^+^ granule cells in one hemisphere, and 3 samples had Prox1^+^ granule cells in both hemispheres. Similar results were also found for calbindin and Ctip2 (Supplementary Table 1). For those sections that did show positive staining of Prox1, calbindin, or Ctip2, the morphology of the dentate gyrus and CA regions was highly abnormal. *Eed-cKO* mice displayed substantial reduction in dentate gyrus area and in the density of Prox1^+^ granule cells. Furthermore, the length of the CA1/2 and CA3 regions were reduced in *Eed-cKO* mice. In contrast, the width of the CA1 region was increased in comparison to controls (Supplementary Fig. 3 AN-AR). However, overall, the areas of both the CA1/2 and CA3 regions were smaller. Moreover, the density of Ctip2^+^ in CA1 region was reduced in *Eed-cKO* mice. Collectively, these results show that *Eed-cKO* mice have distinct hippocampal abnormalities and phenotypic variability between samples and even between hemispheres. *Eed-cHet* samples did not exhibit observable differences compared to controls.

### Glutamatergic neurons exhibit ectopic gene expression in *Eed-cKO* mice

**Fig. 3:**
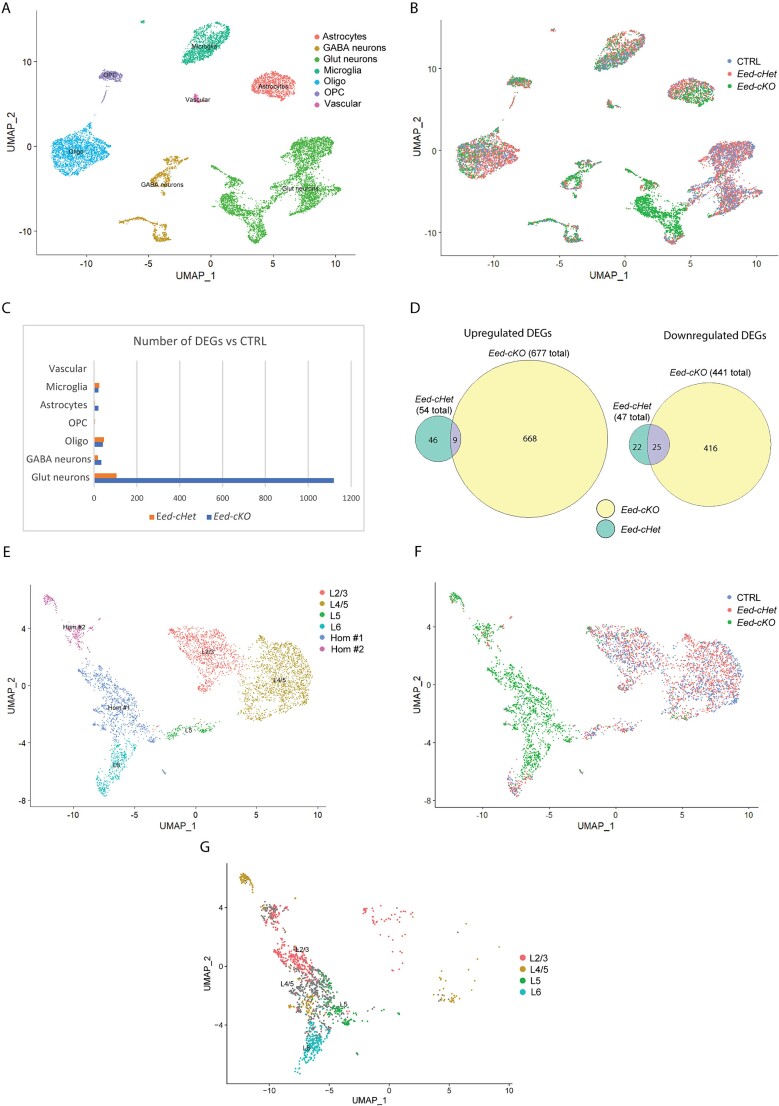
***Eed* regulates gene expression in glutamatergic neurons.** (A) UMAP of combined cells from CTRL, *Eed-cHet*, and *Eed-cKO* samples, revealing that cells cluster by cell type. (B) UMAP of CTRL (red), *Eed-cHet* (blue), and *Eed-cKO* (green) cells, colored by genotype to allow for intergenotype comparison. (C) Total number of DEGs for each major cell type in *Eed-cHet* and *Eed-cKO* mice compared to CTRL. (D) Overlap of DEGs common between both *Eed-cHet* and *Eed-cKO* for upregulated (left) and downregulated (right) genes. (E, G) UMAP visualization of glutamatergic neurons only. Cells are colored according to cluster (E) or genotype (F), demonstrating that CTRL and *Eed-cHet* cells form clusters according to cortical layers, whereas *Eed-cKO* cells form separate clusters. Clusters which could not be categorized into discrete cortical layers using this approach are labeled “Hom #1” and “Hom #2.” (G) Azimuth label transfer to *Eed-cKO* glutamatergic neurons using CTRL annotations as a reference and a transfer score of > 0.75. Oligo = oligodendrocytes, OPC = oligodendrocyte progenitor cells, glut = glutamatergic, micro = microglia.

Our findings are consistent with studies that have previously linked PRC2 function to neural stem cell proliferation and ultimately cortical size (Pereira et al. 2010; Yaghmaeian et al. 2018; Telley et al. 2019; Mora et al. 2022). However, our analysis of laminar marker gene expression hinted at previously unrecognized roles for this complex in mediating cortical glutamatergic cell identity. To interrogate this in more detail, we isolated nuclei from the neocortex of adult CTRL, *Eed-cHet*, and *Eed-cKO* mice and performed single-nucleus RNA-sequencing (snRNA-seq). Following quality control, we sequenced and analyzed 2715, 4660, and 3755 high-quality sequenced nuclei from CTRL, *Eed-cHet*, and *Eed-cKO* mice, respectively (Fig. 3). A Uniform Manifold Approximation and Projection (UMAP) dimension reduction was used to visualize the data. Clustering of cells according to their broad characteristics revealed the expected cortical cell types [astrocytes, GABAergic neurons, glutamatergic neurons, microglia, oligodendrocytes, OPCs, and vascular cells] by the expression of known marker genes in all genotypes (Fig. 3A). However, when we analyzed by genotype, an interesting pattern emerged. While CTRL, *Eed-cHet*, and *Eed-cKO* cells clustered together for most cell types (preoligodendrocytes, oligodendrocytes, astrocytes, microglia, and interneurons), we found that *Eed-cKO* glutamatergic neurons clustered separately from control and *Eed-cHet* cells (Fig. 3B). This suggested that, while most cell types have high similarity in gene expression between genotypes, *Eed-cKO* glutamatergic neurons exhibit divergent gene expression.

This phenomenon was made clearer when we investigated the number of differentially expressed genes (DEGs) (Fig. 3C). Interneurons and microglia are not deleted for *Eed* in this mouse model, and, as anticipated, we did not observe major alterations in gene expression in these cell types. However, astrocytes, a subset of oligodendrocytes, and a subset of preoligodendrocytes, which arise from the dorsal telencephalon and are deleted for *Eed*, all displayed fewer than 50 DEGs each for both *Eed-cHet* and *Eed-cKO* samples, showing that gene expression was not markedly altered in these cell types (Fig. 3C, Supplementary Fig. 4). Collectively, these data suggest that although cortically derived glial cells lack *Eed* in this model, their gene expression profiles are minimally affected in cKO animals.

In stark contrast, glutamatergic neurons displayed substantially altered gene expression in *Eed-cKO* nuclei, with 1,119 DEGs (Fig. 3C). Despite the mild morphological and cellular effects observed in heterozygous animals (Figs. 1 and 2), *Eed-cHet* glutamatergic neurons displayed 105 DEGs (Fig. 3C). Surprisingly, when we compared the cKO versus the cHet DEGs, a relatively small percentage of DEGs were common between *Eed-cHet* and *Eed-cKO* glutamatergic neurons (Fig. 3D). These data suggest that the loss of one *Eed* allele may culminate in a different transcriptional phenotype in comparison to the loss of 2 *Eed* alleles.

Next, glutamatergic neurons were segregated and re-clustered, allowing them to be investigated at greater resolution. By clustering these cells independently from other cell types, we anticipated that they would form further clusters by cortical layer identity. Indeed, both CTRL and *Eed-cHet* glutamatergic neurons formed clusters that could be classified into discrete cortical layers (layers 2/3, 4/5, 5, and 6) based on the top DEGs for each cluster as well as canonical marker genes (Fig. 3E–F, Supplementary Table 2). However, nuclei from *Eed-cKO* glutamatergic neurons did not form readily identifiable clusters based on these markers, instead resolving into 3 abnormal clusters. To better annotate the *Eed-cKO* clusters, we utilized Azimuth (Hao et al. 2021), a single cell profile transfer tool which mapped cell annotations from the CTRL clusters to glutamatergic nuclei in the *Eed-cKO* data set. In addition, a transfer label score threshold of greater than 0.75 was used to allow visualization of only the strongest transfer labels. While *Eed-cKO* nuclei exhibited abnormal gene expression, the Azimuth tool enabled their classification into layers most similar to 2/3, 4/5, 5, and 6 (Fig. 3G). Altogether, this demonstrates that although cortical lamination is relatively intact in *Eed-cKO* mice, the gene expression profile of *Eed-cKO* neurons is highly abnormal.

**Fig. 4:**
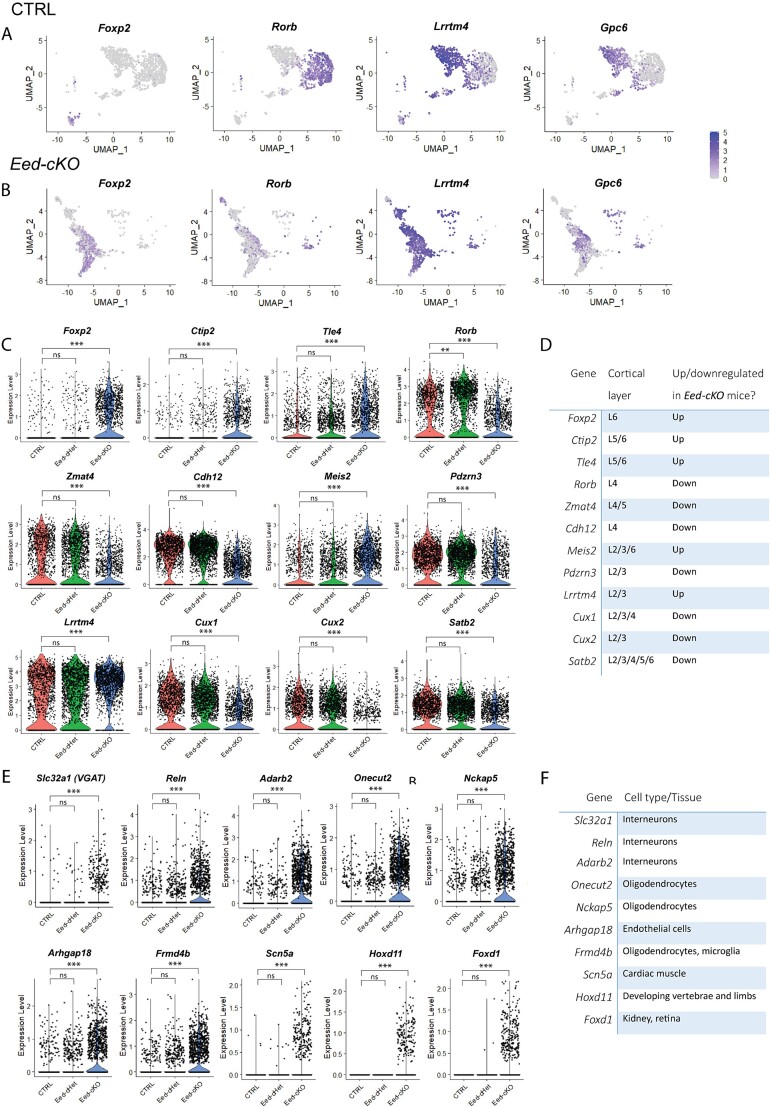
**Glutamatergic neurons exhibit aberrant gene expression in *Eed-cKO* mice.** (A, B) UMAPs showing expression of cortical layer markers *Foxp2*, *Rorb, Lrrtm4*, and *Gpc6* (denoting layer 6, 4, 2/3, and 2/3, respectively) in CTRL (A) and *Eed-cKO* (B) glutamatergic neurons, allowing visualization of how these markers are expressed across clusters. Dark blue represents cells with high expression of these genes. In CTRL cells, markers are largely expressed in a single cluster, whereas in *Eed-cKO* cells they are broadly expressed across clusters. (C) Violin plots showing examples of dysregulated gene expression of cortical layer markers in *Eed-cKO* neurons. (D) List of which cortical layers the genes in (C) are normally expressed in. Also listed is whether these genes were up or downregulated in *Eed-cKO* neurons. (E) Examples of genes that were ectopically expressed in *Eed-cKO* glutamatergic neurons. (F) A table showing examples of what cell types or tissues the genes in (E) are usually expressed in. See also Supplementary Fig. 5 for gene ontology analysis.

Indeed, when we examined the top DEGs in *Eed-cKO* glutamatergic neurons, we found that many were genes that normally have cortical layer-specific expression. For example, *Foxp2* is usually expressed only in layer 6 cortico-thalamic projection neurons and a small population of layer 5 neurons (Ferland et al. 2003). Control and *Eed-cHet* neurons had a single cluster strongly expressing *Foxp2*. However, *Foxp2* was expressed throughout many *Eed-cKO* clusters. Similar results were found for other cortical layer markers, including *Lrrtm4* (L2/3), *Gpc6* (L2/3), and *Rorb* (L4) (Fig. 4A–B). Not only were many cortical layer markers spread across clusters, but they were also highly dysregulated in *Eed-cKO* neurons. For example, the layer 2/3 gene *Lrrtm4,* and layer 5/6 genes *Foxp2*, *Ctip2*, and *Tle4* were upregulated in mutant nuclei. Conversely, the layer 2/3 genes *Cux1* and *Pdzrn3*, and layer 4 genes *Zmat4* and *Cdh12* were downregulated in the mutant (Fig. 4C). Supporting the notion of abnormal cellular identity, *Eed-cKO* neurons also showed ectopic expression of many genes that are not normally expressed in glutamatergic neurons, including genes that are normally expressed in interneurons, or glia. Moreover, we also revealed the expression of factors normally absent from the CNS, including *Hoxd11* and *Foxd1* (Fig. 4E–F), the former that we validated with immunofluorescent staining and real-time quantitative PCR (Supplementary Fig. 5).

In line with this, GO enrichment analysis identified that neuron-specific processes were significantly enriched in *Eed-cKO* mice (Supplementary Fig. 6). In *Eed-cKO* neurons, top enriched biological process included terms such as synaptic organization, cell junction assembly, neurotransmitter transition, membrane potential, transmembrane transport, axonogenesis and dendrite development. Top enriched molecular functions involved transmembrane transport, ion channels, and actin binding, and the synapse was the top implicated cell compartment. *Eed-cHet* neurons shared similarly enriched GO terms. Collectively, these transcriptomic analyses are consistent with the absence of H3K27 methylation during development culminating in abnormal glutamatergic neuron identity within the adult cerebral cortex.

### *Eed-cKO* pyramidal neurons display morphological deficits

**Fig. 5:**
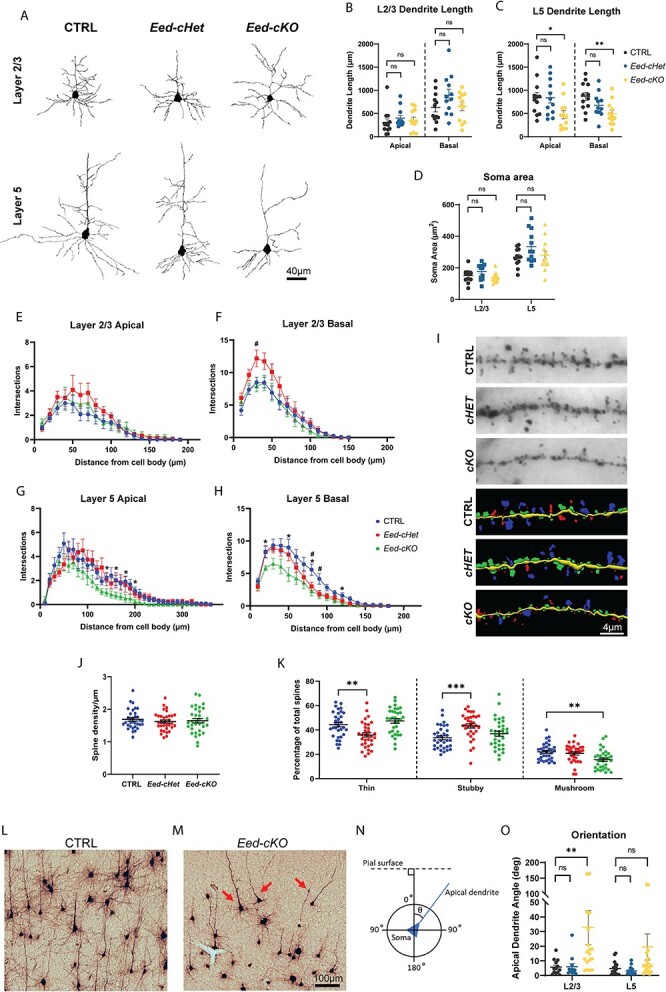
***Eed-cKO* pyramidal neurons have morphological deficits.** Golgi–Cox analysis of CTRL, *Eed-cHet*, and *Eed-cKO* pyramidal neurons in layers 2/3 and 5 of the motor cortex. (A) Examples of pyramidal neuron morphology in layer 2/3 and 5 of the motor cortex in CTRL, *Eed-cHet*, and *Eed-cKO* mice. (B–C) Measurement of apical and basal dendrite length in layer 2/3 (B) and 5 (C) neurons. (D) Measurement of soma area in layer 2/3 and 5 pyramidal neurons. (E–H) Sholl analysis of dendrite complexity, which counts the number of dendrite branches in 10 μm intervals from the soma. # = *P* < 0.05 (*Eed-cHet*), ^*^ = *P* < 0.05 (*Eed-cKO*), 2-way ANOVA (E–H). *n* = 12 neurons from 4 mice per genotype. (I-K) Analysis of spine morphology. (I) Examples of imaged (top) and digitally traced (bottom) dendrites with dendritic spines in CTRL, *Eed-cHet*, and *Eed-cKO* pyramidal neurons. (J) Density of dendritic spines was measured. Each point represents an individual dendrite. (K) The percentage of thin, stubby, and mushroom shaped spines was also measured. Each point represents the mean of all spines on an individual dendrite. ^*^^*^ = *P* < 0.01, ^*^^*^^*^ = *P* < 0.001, ^*^^*^^*^^*^ = *P* < 0.0001, one-way ANOVA with Dunnett’s multiple comparisons. *n* = 33 dendrites from 11 neurons per genotype across 4 CTRL, 4 *Eed-cHet*, and 3 *Eed-cKO* mice. (L–O) Analysis of apical dendrite orientation. (L, M) Examples of pyramidal neuron orientation in CTRL (L) and *Eed-cKO* (M) cortex. In the CTRL cortex, pyramidal neurons were orientated parallel to each other, however in the *Eed-cKO* cortex, there were several pyramidal neurons which were not parallel to each other (arrows). (N) Schematic of how orientation was measured. The angle of the apical dendrite was measured relative to the pial surface. (O) Quantification of apical dendrite orientation. ^*^^*^ = *P* < 0.01, ns = not significant, Kruskal-Wallis test with Dunn’s multiple comparisons. Layer 2/3: *n* = 12 CTRL, 12 *Eed-cHet*, and 17 *Eed-cKO* neurons from 4 mice per genotype. Layer 5: *n* = 24 CTRL, 15 *Eed-cHet*, and 23 *Eed-cKO* neurons from 4 mice per genotype.

Are these aberrant cellular identities mirrored in abnormal neuronal morphology? The enrichment of GO terms associated with axonogenesis and dendrite development supports this notion. To investigate this, we assessed dendritic architecture in glutamatergic neurons of the motor cortex using Golgi–Cox staining (Fig. 5A). Dendritic length and arborization in mutant neurons within layer 2/3 was similar to the CTRL. However, *Eed-cKO* neurons in layer 5 had reduced dendrite length (Fig. 5B–C) and arborization (Fig. 5B–C). These changes may result from cell-intrinsic dendritic development defects or from the smaller cortex of *Eed-cKO* mice, as dendrites are unable to project their normal distances due to the reduced cortex size. Remarkably, *Eed-cHet* neurons had a distinct morphology from *Eed-cKO* neurons. Although layer 5 basal dendrites showed decreased arborization, akin to *Eed-cKO* neurons (Fig. 5H), *Eed-cHet* neurons in layer 2/3 had an increased number of branches in basal dendrites in comparison to the CTRL (Fig. 5F). Next, we analyzed dendritic spines in layer 2/3 pyramidal neurons (Fig. 5I–M). Spine density was not significantly altered in *Eed-cHet* or *Eed-cKO* mice compared to controls (Fig. 5J). Spines were then classified into 3 groups based on their morphology: thin, stubby, or mushroom. In general, mushroom spines are mature with strong synaptic connections, thin spines are less mature but more dynamic, and stubby spines are thought to be immature, transitory structures (Berry and Nedivi 2017). We found that *Eed-cKO* mice had a lower proportion of mushroom spines, whereas *Eed-cHet* mice had a lower proportion of thin spines, and a higher proportion of stubby spines, compared to controls (Fig. 5K). Overall, these results suggest that *Eed-cHet* and *Eed-cKO* neurons have different deficits in spine maturation.

During this analysis, we also observed that a small proportion of *Eed-cKO* pyramidal neurons were misoriented (Fig. 5M). To quantify this, the angle of the primary apical dendrite was measured relative to the pial surface (Fig. 5L–O). There was no difference in orientation in *Eed-cHet* neurons compared to CTRL, or in layer 5 *Eed-cKO* neurons. However, in layer 2/3 *Eed-cKO* neurons, the median apical dendrite angle was higher when compared to control neurons (Fig. 5O). Hence, the apical dendrites of *Eed-cKO* neurons tended to be less perpendicular to the pial surface than in control neurons. This suggest that *Eed-cKO* pyramidal neurons might have deficits in apical dendrite guidance, causing abnormal neuron orientation. Overall, these results demonstrate that both *Eed-cHet* and *Eed-cKO* mice have deficits in dendritic arborization within pyramidal neurons, with the divergent dendritic phenotypes consistent with the limited overlap in DEGs between *Eed-cHet* and *Eed-cKO* neurons.

### *Eed-cKO* mice have deficits in global white matter connectivity

**Fig. 6:**
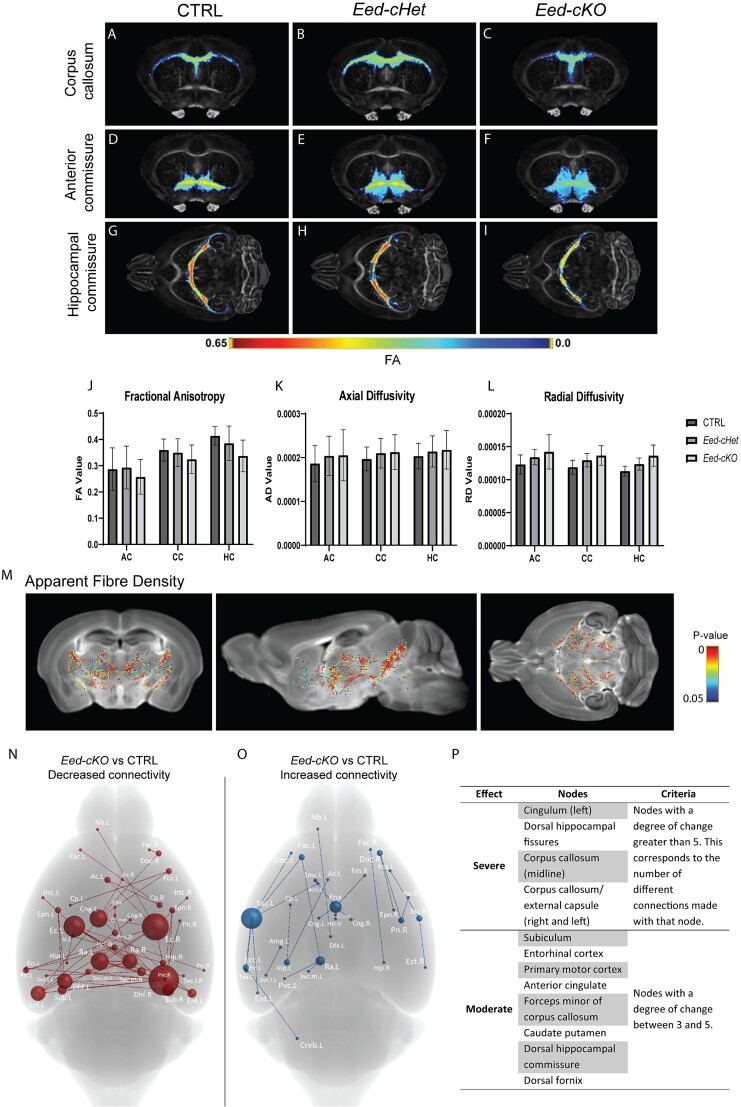
***Eed-cKO* mice have deficits in global white matter connectivity.** (A–L) DTMRI metrics in the forebrain commissures of CTRL, *Eed-cHet*, and *Eed-cKO* mice. (A–R) Fractional anisotropy of the corpus callosum (A–C), anterior commissure (D–F), and hippocampal commissure (G–I) in CTRL, *Eed-cHet*, and *Eed-cKO* brains. There was no significant difference between genotypes. (J–L) Measurement of fractional anisotropy (J), axial diffusivity (K), and radial diffusivity (L) in the anterior commissure (AC), corpus callosum (CC), and hippocampal commissure (HC). There was no significant difference between genotypes. (M) Significant changes in apparent fiber density were observed in *Eed-cKO* mice compared to CTRL. (N–P) Connectome analysis of *Eed-cKO* brains. Graphic of key brain regions (“nodes”) which have decreased (N) or increased (O) connections to other regions in *Eed-cKO* mice. (P) A list of severely and moderately affected nodes in *Eed-cKO* mice. *Eed-cHet* brains are not displayed as there were no significant differences. *n* = 7 CTRL, 8 *Eed-cHet*, 5 *Eed-cKO*.

Our findings of compromised neuronal structures (Fig. 5) and forebrain commissures (Fig. 1) suggested that cortical connectivity may also be aberrant in *Eed-cKO* mice. To investigate this, we used DTMRI to reconstruct white matter tractography of the brain. First, we used DTMRI to examine metrics that provide information about the microstructural properties of white matter—fractional anisotropy (FA), axial diffusivity (AD), and radial diffusivity (RD) (Basser et al. 1994). These metrics can be impacted by various changes in the tissue, such as alterations in tissue density, myelination, or composition of the extracellular matrix. Surprisingly, there was no significant difference in FA, RD, or AD in the corpus callosum, hippocampal commissure, or anterior commissure in *Eed-cHet* or *Eed-cKO* mice (Fig. 6A–L). Voxel-based morphometry detected changes in FA in several gray matter regions in the *Eed-cKO*, including the dentate gyrus and cortical plate (Supplementary Fig. 7). Nevertheless, the lack of changes in FA, RD, or AD in the commissures suggest that white matter integrity is not largely compromised in *Eed-cHet* or *Eed-cKO* mice. Interestingly, however, we detected significant reductions in apparent fiber density (Raffelt et al. 2017) in *Eed-cKO* mice, particularly in the internal capsule, hippocampal commissure, and in white matter tracts connecting the thalamus and globus pallidus (Fig. 6M). By contrast, fiber cross section (Raffelt et al. 2017) was not affected. Together, this suggests that although the size of the corpus callosum and hippocampal commissure is dramatically reduced (Fig. 1), the structure of the tracts present in *Eed-cKO* mice are relatively normal, albeit less dense.

To take a more comprehensive approach to analyze connectivity, we next performed a 3D reconstruction of the neural connections in the brain, allowing global connectivity to be investigated (Zalesky et al. 2010). There was no significant change in the connectome detected between *Eed-cHet* and CTRL mice. However, *Eed-cKO* mice had numerous regions with significantly altered connectivity compared to CTRLs. Decreased connectivity was most common (Fig. 6N), but there were also regions with increased connectivity (Fig. 6O). The most severely affected regions included the cingulum, dorsal hippocampal fissures, and corpus callosum. The subiculum, entorhinal cortex, primary motor cortex, anterior cingulate cortex, caudate putamen, and dorsal fornix were also moderately affected (Fig. 6P). We observed that many of the decreased connections involved interhemispheric connections, which was likely related to the smaller corpus callosum and hippocampal commissure.

### Loss of *Eed* results in mild behavior alterations in the adult

**Fig. 7:**
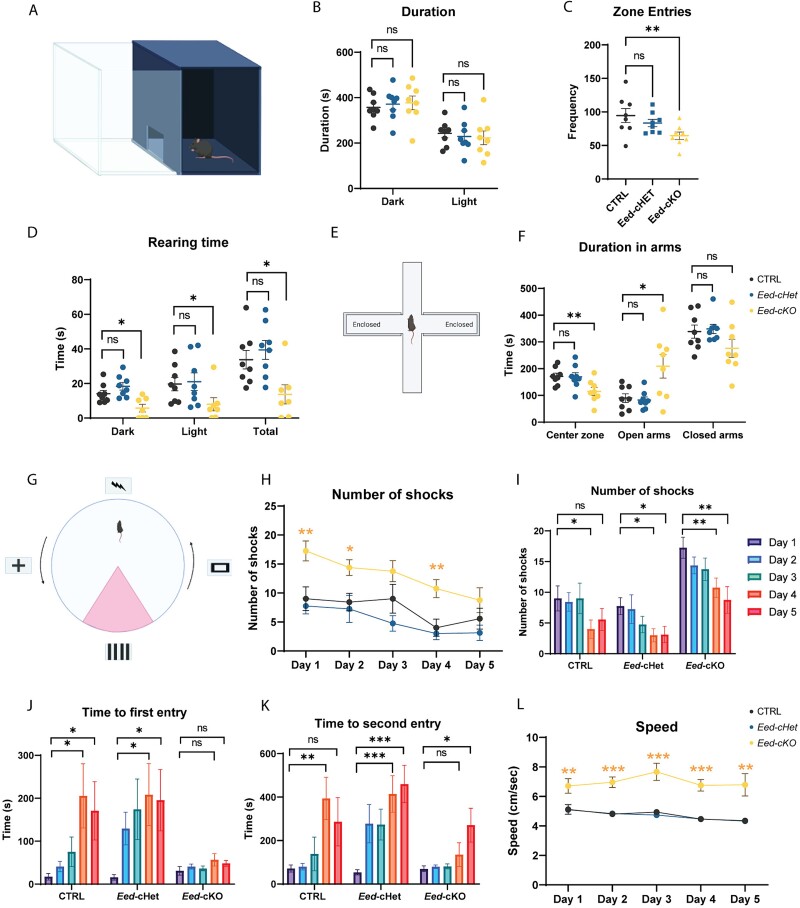
**Loss of *Eed* results in mild behavior alterations in the adult.** (A–D) Light/dark box test. (A) Schematic of the light/dark box arena. (B–D) Duration in outer and inner zone (B), number of zone entries (C), and time spent rearing (D) was measured. There was no significant difference in time spent on other activities (ambulatory, stereotypic, jumping, resulting; data not shown). (E–F) Elevated plus maze test. (E) Schematic of the elevated plus maze arena. (F) Duration in center zone, open arms, and closed arms was measured. There was no significant difference in frequency in arms, latency to arms, distance moved, average speed, duration not moving, or frequency of head dips (data not shown). (G–L) Active place avoidance test. (G) Schematic of the active place avoidance arena. (H) Number of shocks is displayed in graphs for inter-genotype (H) and intra-genotype (I) comparison. Time to first (J) and second (K) shock and average speed (L) are also shown. In (H and L), asterisks indicate statistical significance for *Eed-cKO* compared to CTRL. ^*^^*^^*^ = *P* < 0.001, ^*^^*^ = *P* < 0.01, ^*^ = *P* < 0.05, one-way ANOVA. *n* = 8 CTRL, 8 *Eed-cHet*, 8 *Eed-cKO* (B, C, F) or 7 CTRL, 8 *Eed-cHet*, 8 *Eed-cKO* (H–L).

Given the severe morphological phenotypes in *Eed-cKO* mice, as well as the distinct phenotypes within the *Eed-cHet*, we hypothesized that these mice would exhibit reduced performance in cortically-related behavioral tests. To investigate this, we first performed the light–dark box test, which is used to investigate anxiety vs exploratory behaviors (Fig. 7A–D) (Bourin and Hascoët 2003). Interestingly, despite the changes in connectivity observed between limbic structures implicated in anxiety (Fig. 6N–P), *Eed-cHet* and *Eed-cKO* mice did not spend more time in the light or dark component compared to controls (Fig. 7B), although *Eed-cKO* mice did have reduced frequency in the number of times they moved between compartments (Fig. 7C). Similarly, we did not observe any anxiety-related behaviors in *Eed-cHet* or *Eed-cKO* mice in the open field test (Supplementary Fig. 8A–F) (Seibenhener and Wooten 2015). Anxiety/exploratory behaviors were further investigated with the elevated plus maze assay (Fig. 7E–F) (Walf and Frye 2007). Surprisingly, *Eed-cKO* mice spent more time in the open arms than controls, suggesting that *Eed-cKO* mice may have less fear of open spaces than control mice, or that they are incapable of perceiving the danger (Fig. 7F).

Next, we assessed cognitive flexibility with the cognitively challenging dynamic strategy-shifting test. Here, mice are tasked to press a lever based on various visual and nonvisual cues for a food reward. Despite the severe range of phenotypes at a cellular and molecular level in the *Eed-cKO* mice, there was no significant difference between genotypes, suggesting that cognitive flexibility was not compromised in *Eed-cHet* or *Eed-cKO* mice (Supplementary Fig. 9). *Eed-cHet* and *Eed-cKO* mice also do not display any deficits in sociability, as there were no significant differences in *Eed-cHet* or *Eed-cKO* mice in the 3-chambered social interaction test (Supplementary Fig. 8G–M) (Kaidanovich et al. 2011).

The severe hippocampal phenotype observed in cKO animals (Figs. 1 and 2) led us to postulate that learning and memory may be abnormal in our cKO mice. To investigate this, we used an active place avoidance paradigm (Fig. 7G–L, Supplementary Fig. 8K–O) (Stuchlík et al. 2013). The CTRL and *Eed-cHet* mice both received fewer shocks on later days of the trial in comparison to the first day of the trial, indicative of learning. To our surprise, although *Eed-cKO* animals received consistently more shocks each day over the 5-day protocol, they also exhibited learning, as evidenced by fewer shocks per day on day 5 compared to day 1 (Fig. 7I). Closer analysis revealed that *Eed-cKO* mice did not exhibit a significant change in the time to the first shock each day—a proxy for long term memory—(Fig. 7J). However, they did show improvements in short term memory, as measured by the time to the second entry to the shock zone (Fig. 7K). The speed of the *Eed-cKO* animals was also increased over all 5 d of the trial (Fig. 7L). Again, these data point to an unexpected flexibility in our *Eed-cKO* mice; despite having severe hippocampal abnormalities, these mice were still able to learn in a hippocampal-dependent behavioral task. This is in stark contrast to other models that have similar hippocampal deficits and reduced cortical size, such as mice lacking *Usp9x* (Johnson et al. 2020).

## Discussion

Here we have characterized the adult effects of embryonic PRC2 loss-of-function in the dorsal telencephalon. As anticipated from previous studies, adult *Eed-cKO* mice exhibit microcephaly, with significantly smaller cortical structures including the cortical plate, corpus callosum, and hippocampus. Our studies reveal several novel findings. Firstly, we show that glutamatergic neurons are preferentially affected in the absence of H3K27 methylation, whereas other cellular populations derived from the dorsal telencephalon, such as astrocytes and oligodendrocytes, appear relatively normal. Secondly, we reveal that cellular identity within glutamatergic *Eed-cKO* neurons is abnormal, as are aspects of dendritic structure. Thirdly, we reveal that heterozygous and homozygous mice display divergent phenotypes. Finally, we reveal that, despite a range of molecular, cellular, and structural cortical deficits, *Eed-cKO* mice function with a surprising level of effectiveness in a range of behavioral tasks, some of which are cognitively challenging.

One of the main findings of this work is that *Eed-cKO* glutamatergic neurons have highly dysregulated expression of neuron-specific genes and the ectopic expression of interneuron, glial, and peripheral genes. Similar findings of abnormal gene identity profiles have been identified in the hypothalamus of *Eed-cKO* mice (Yaghmaeian Salmani et al. 2022), indicating that PRC2 may regulate neuron identity in different regions of the brain. Surprisingly, gene expression of cortical oligodendrocytes and astrocytes was not strongly affected, indicating that *Eed*, and consequently PRC2, does not play a major role in regulating gene expression in these cell types. Whether PRC2 function is similarly required for the specification of interneurons remains an open question; the use of different Cre drivers in future will be needed to investigate this possibility.

Gene ontology analysis indicated that numerous neuronal functions were altered in both *Eed-cHet* and *Eed-cKO* mice, such as synapse function, axonogenesis, and dendrite development. In line with this, Golgi–Cox analyses identified deficits in both *Eed-cHet* and *Eed-cKO* mice in dendrite arborization and dendritic spine morphology, indicating that synaptic connections were abnormal. Interestingly, we also found that a small proportion of *Eed-cKO* pyramidal neurons were misoriented, which may indicate that *Eed* plays role in growth cone guidance. This notion is further supported by the connectome analysis, showing both decreased and increased connections between several brain regions. The connectome changes may underlie the effects upon some of the brain regions indirectly affected by *Emx1-iCre* deletion of *Eed*, such as the thalamus, which is highly interconnected to the neocortex and therefore may be smaller due to reduced reciprocal connections to the neocortex (Antón-Bolaños et al. 2018). In future, electrophysiological studies could be useful to elucidate neural function in the absence of *Eed* in more detail.

The behavioral analysis revealed several unexpected results. Considering their cortical and hippocampal phenotypes, *Eed-cKO* mice performed surprisingly well at many tasks, including tasks involving exploration, social interaction, learning and memory, and cognitive flexibility. These tasks are cognitively complex and involve multiple cortical and limbic structures, many of which are severely diminished in *Eed-cKO* mice. Could subcortical structures be compensating for the diminished cortical structures in *Eed-cKO* mice? Functional MRI may provide further insight into this, as could a broader screen of neuronal activity through the investigation of immediate early gene expression, such as *c-fos* (Chung 2015). Additionally, future studies comparing this model with other mouse models with similar structural deficits of the cortex and hippocampus that have markedly worse behavioral outcomes may help to elucidate the structural and molecular mechanisms underlying behavioral plasticity.

In *Eed-cHet* mice, we did not observe any major alterations in the structure, cytoarchitecture, or connectivity in the cortex, or in behavior. However, *Eed-cHet* mice did have alterations in glutamatergic neuron gene expression and pyramidal neuron morphology. As such, it appears that while the phenotype of *Eed-cHet* mice is subtle, loss-of-function of one allele of *Eed* is enough to cause deficits in cortical development. Remarkably, *Eed-cHet* mice often had a divergent phenotype to *Eed-cKO* mice. For instance, only a very small proportion of DEGs overlapped between *Eed-cHet* and *Eed-cKO* neurons. Moreover, *Eed-cHet* pyramidal neurons had increased dendritic arborization in upper layers, which *Eed-cKO* pyramidal neurons did not show. *Eed-cHet* dendrites also had a higher proportion of stubby spines whereas *Eed-cKO* dendrites had a lower proportion of mushroom spines. Collectively, this suggests that that loss of one allele (*Eed-cHet*) vs 2 alleles (*Eed-cKO*) of *Eed* may have different epigenetic and gene expression consequences, pointing to a dose-sensitivity of the PRC2 complex. The heterozygous phenotypes observed in our *Eed-cHet* animals may have bearing upon the role of PRC2 in humans, where the 3 key PRC2 genes *EED*, *EZH2*, and *SUZ12* are all haploinsufficient (Lek et al. 2016; Karczewski et al. 2020). Moreover, the human Overgrowth and Intellectual Disability disorders Cohen–Gibson syndrome, Weaver syndrome and Imagawa-Matsumoto syndrome are linked to heterozygous gain-of-function mutations to *EED*, *EZH2*, or *SUZ12* (Tatton-Brown et al. 2011; Cohen and Gibson 2016; Imagawa et al. 2018). These syndromes present with neurological defects, which involve delayed speech and psychomotor development, and intellectual disability. MRI analysis has not been widely conducted on these patients, but in the few cases where it has been used it has shown anatomical defects, including polymicrogyria and white matter volume loss (Cohen et al. 2016; Cooney et al. 2017). In the future, a more comprehensive investigation patients using MRI, coupled with the generation and characterization of mice with patient-specific mutations, may permit an improved understand of these conditions. A scenario is thus emerging where half-dose, no-dose, and neo/hyper-morphic genetic defects in PRC2 genes all negatively affect mammalian brain development in diverse ways.

## Supplementary Material

Supplementary_figures_tables_and_methods_bhae268
