# Supplementary material for: Polycomb repressive complex 2 is critical for mouse cortical glutamatergic neuron development
Source: Cereb Cortex. 2024 Jul 3;34(7):bhae268. doi: 10.1093/cercor/bhae268 (PMC11221884; doi:10.1093/cercor/bhae268)
Supplement: Supplementary_figures_tables_and_methods_bhae268 [file supplementary_figures_tables_and_methods_bhae268.docx]

# Supplementary Figures


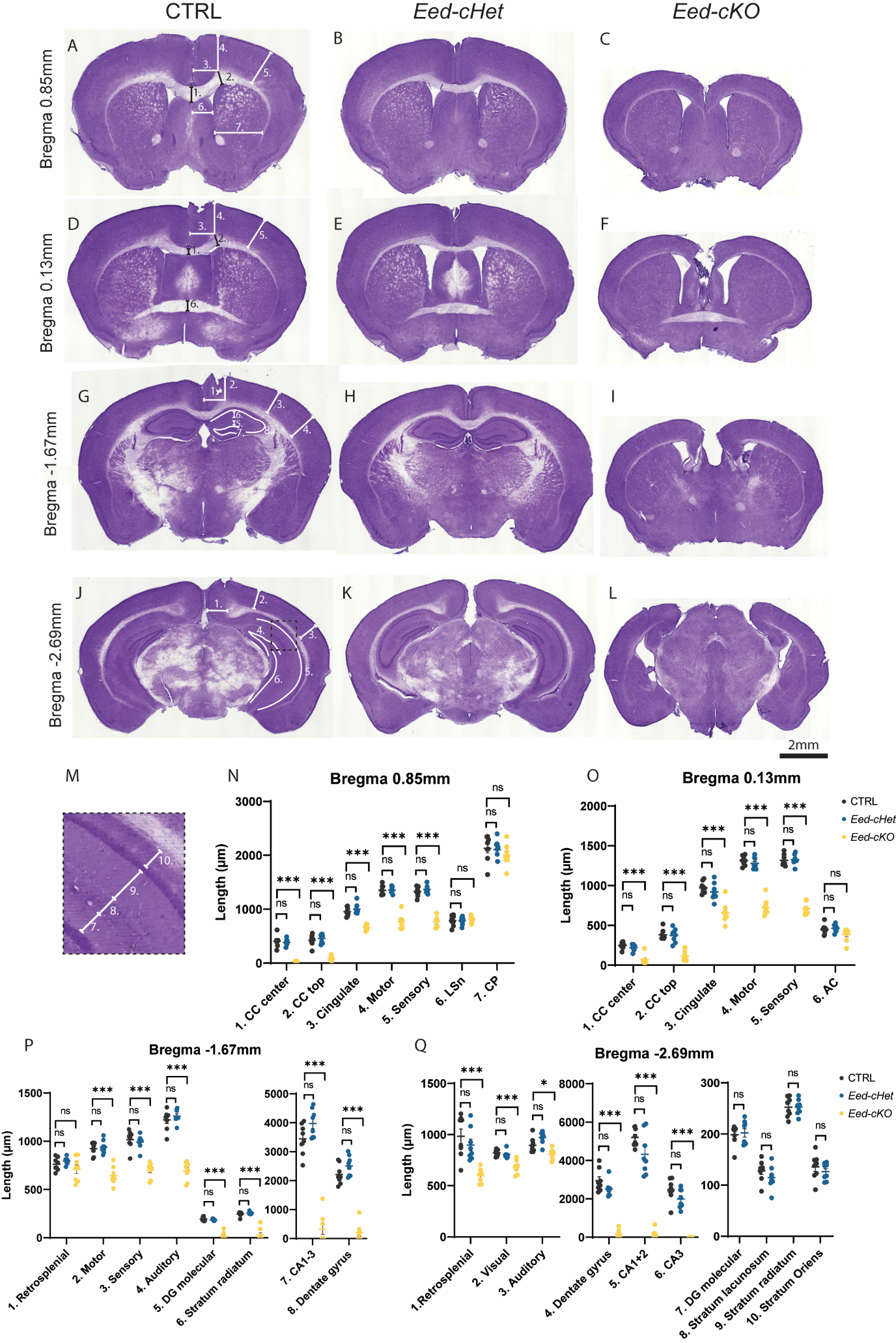


**Supplementary Figure 1 – Morphometric analysis of *Eed-cHet* and *Eed-cKO* mice at Bregma 0.85 mm, 0.13 mm, -1.67 mm, and -2.69 mm**

Haematoxylin-stained coronal sections of CTRL, *Eed-cHet*, and *Eed-cKO* brains at Bregma 0.85 mm (A-C, N), Bregma 0.13 mm (D-F, O), Bregma -1.67 mm (G-I, P), and Bregma -2.69 mm (J-L, Q). Annotations on (A, D, G, J, M) indicate how regions of interest were measured. CC = corpus callosum, LSn = lateral septal nucleus, AC = anterior commissure, HC = hippocampal commissure. *** = p<0.001, ns = not significant, one-way ANOVA. n = 8 CTRL, 8 *Eed-cHet*, 8 *Eed-cKO*.


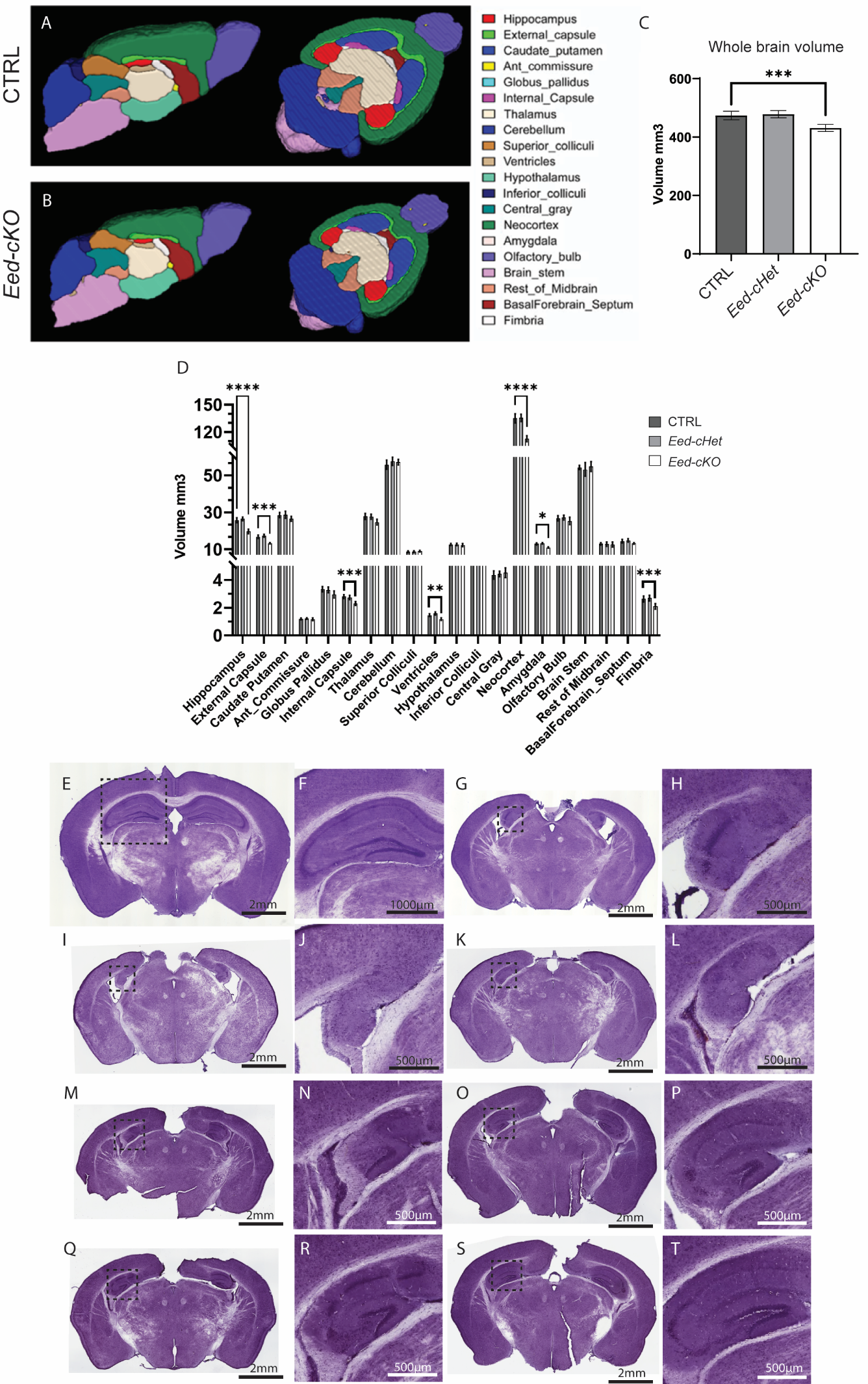


**Supplementary Figure 2 – Structural MRI analysis and variation of the *Eed-cKO* hippocampal phenotype**

(A-D) Structural MRI analysis of CTRL, *Eed-cHet*, and *Eed-cKO* mice. (A, B) Representative images of 3D reconstruction from MRI scans of control (A) and *Eed-cKO* (B) brains. (C) Measurement of whole brain volume. (D) Volume measurements of major brain regions. * = p<0.005, ** = p<0.001, ns = not significant. n = 7 CTRL, 8 *Eed-cHet*, 5 *Eed-cKO*. (E-T) Haematoxylin sections of one control (E, F) and 7 *Eed-cKO* biological replicates (G-T) demonstrates the variation of hippocampal phenotype in *Eed-cKO* brains. Panels (F, H, J, L, N, P, R, T) show zoomed-in images of the hippocampus of (E, G, I, K, M, O, Q, S), respectively. Panel H shows a *Eed-cKO* hippocampus with a structure resembling a CA region (yellow arrow), but no apparent dentate gyrus. Panel J and L show *Eed-cKO* brains which do not have any clear hippocampal structures. Panel N shows a *Eed-cKO* hippocampus with a structure resembling a dentate gyrus (orange arrow), but no apparent CA1-3 region. Panels P, R, and T show a *Eed-cKO* hippocampus which has both a CA region and dentate gyrus (yellow and orange arrow respectively), although they were clearly diminished compared to the control (B).


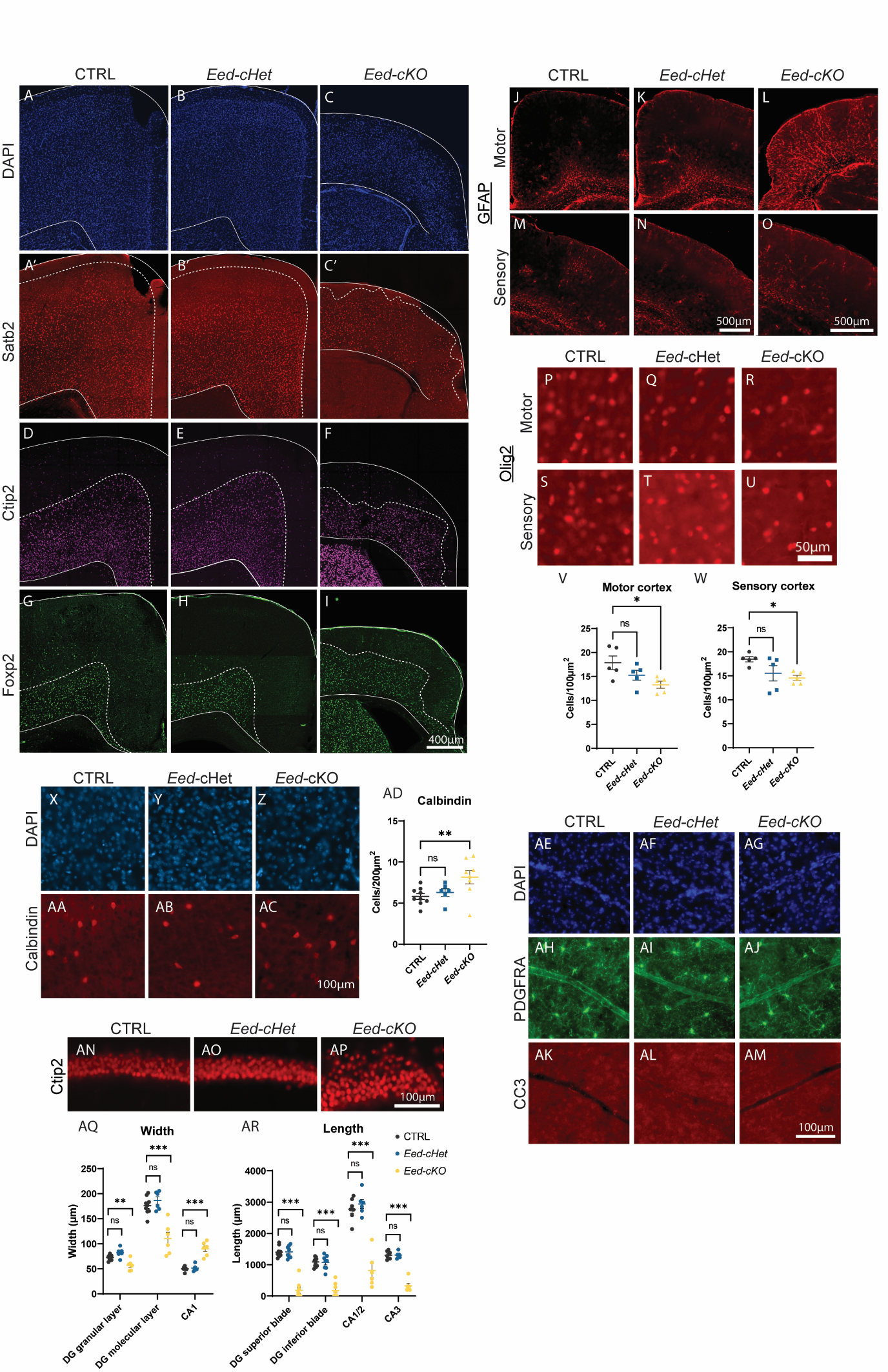


**Supplementary Figure 3 – Additional immunofluorescent analysis of *Eed-cHet* and *Eed-cKO* brains**

(A-I) Immunofluorescence staining of DAPI (A-C), Satb2 (A’-C’), Ctip2 (D-F), and Foxp2 (G-I) of the CTRL, *Eed-cHet*, and *Eed-cKO* motor/cingulate cortex in adult brains. DAPI and Satb2 were co-stained, whereas Ctip2 and Foxp2 were stained from separate sections. Dashed lines represent the border between positive and negative layers for each marker. In CTRL and *Eed-cHet* samples, the border was an organised, straight line, whereas in *Eed-cKO* samples, the border was irregular and sometime ambiguous, indicating that cortical lamination was disorganised in *Eed-cKO* mice. (J-O) Immunofluorescence stains of GFAP in the motor (J-L) and sensory (M-O) cortices of CTRL, *Eed-cHet*, and *Eed-cKO* adult mice. Compared to controls, *Eed-cKO* mice had increased expression of GFAP in the motor/cingulate cortex (L), but not in the sensory cortex (O). (P-W) Immunofluorescence staining of Olig2 in the motor (P-R) and sensory (S-U) cortex of CTRL, *Eed-cHet*, and *Eed-cKO* adult mice. Olig2^+^ cells were counted in the motor cortex (V) and sensory cortex (W). (X-AD) Immunofluorescence stain of DAPI (X-Z) and calbindin (AA-AC) in the sensory cortex of CTRL, *Eed-cHet*, and *Eed-cKO* adult mice. Calbindin^+^ cells were counted in the middle layers of the sensory cortex (AD). (AE-AM) Immunofluorescence staining of DAPI (AE-AG), PDGFRA (AH-AJ) and CC3 (AK-AM) to investigate blood vessel integrity. PDGFRA labels vascular endothelial cells and oligodendrocyte precursor cells and CC3 labels cell death. No abnormalities were observed in blood vessel integrity or cell death. (AN-AR) Supplementary data of immunofluorescence stains of Prox1, calbindin, and Ctip2 in the hippocampus of CTRL, *Eed-cHet*, and *Eed-cKO* adult mice (presented in Figure 2). (AN-AP) Zoomed in image of the Ctip2^+^ CA1 region in CTRL, *Eed-cHet*, and *Eed-cKO* samples shows this region in greater detail. (AQ) The width of the dentate gyrus (DG) granular and molecular layers was measured, as well as the width of the CA1 region. (AR) The length of the dentate gyrus superior and inferior blades, CA1/2, and CA3 regions were measured. *** = p<0.001, ** = p<0.01, * = p<0.05, ns = not significant, one-way ANOVA. n = 5 CTRL, 5 *Eed-cHet*, 5 *Eed-cKO* (V-W); n = 9 CTRL, 6 *Eed-cHet*, 8 *Eed-cKO* (AD); n = 10 CTRL, 8 *Eed-cHet*, 8 *Eed-cKO* (AQ-AR).


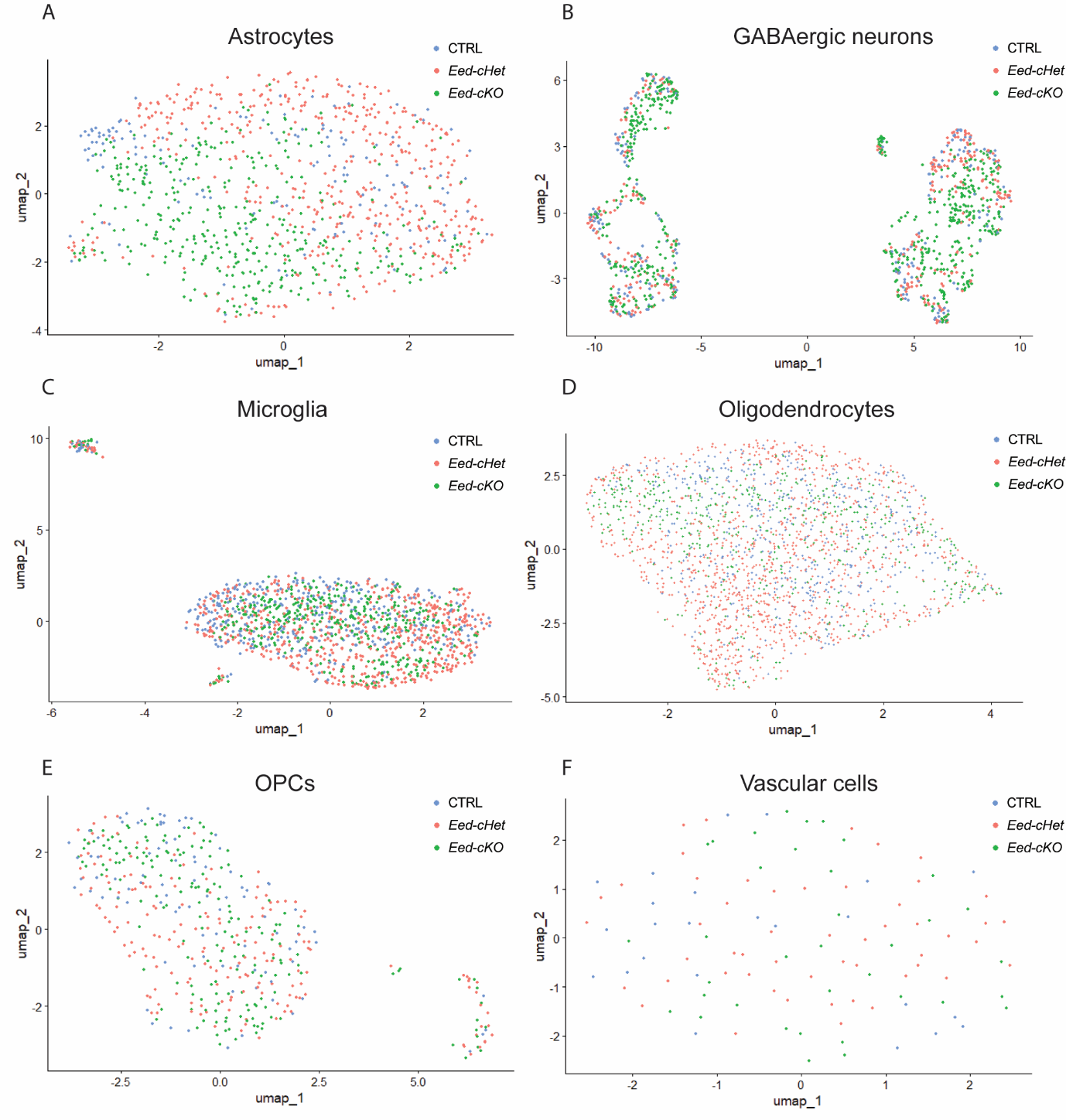


**Supplementary Figure 4 – snRNAseq analysis of other cell types**

UMAPS of astrocytes (A), GABAergic neurons (B), microglia (C), oligodendrocytes (D), OPCs (E), and vascular cells (F). Each dot represents a CTRL (red), *Eed-cHet* (blue), or *Eed-cKO* (green) cell. There were no major differences between genotypes in these cell types.


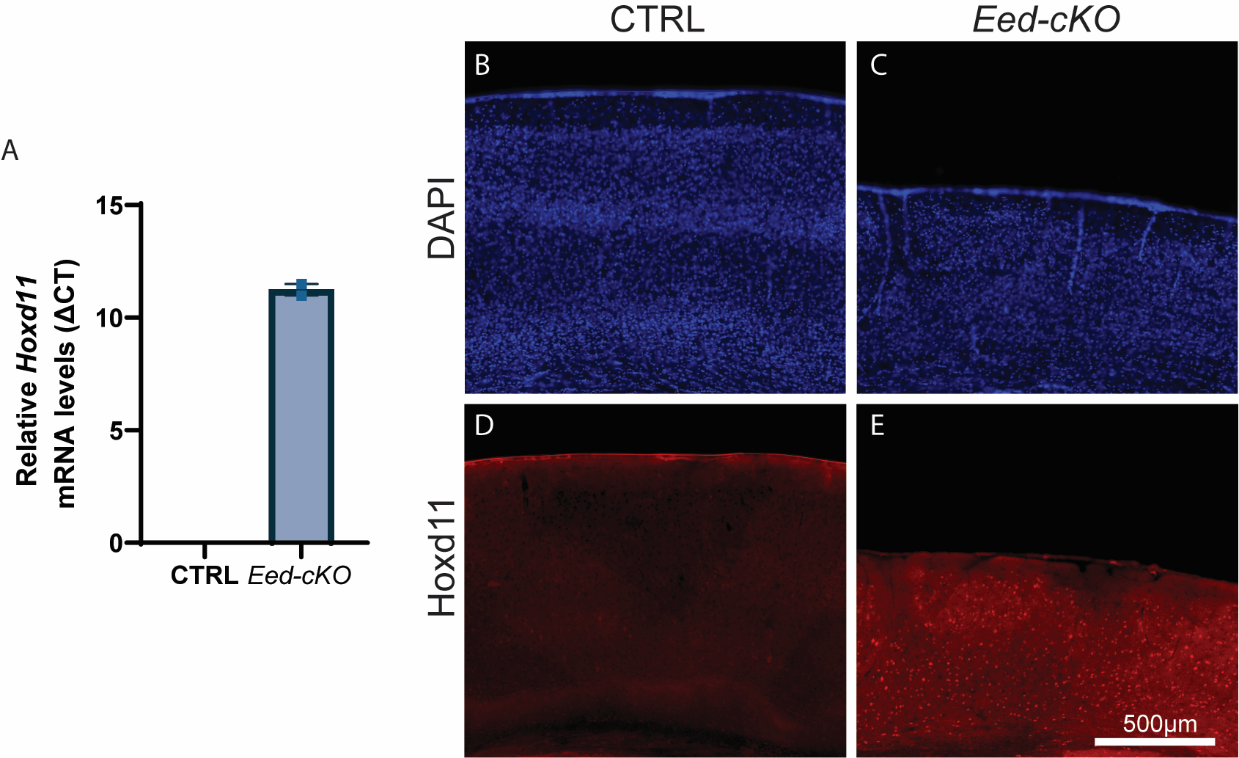


**Supplementary Figure 5 – Validation of ectopic Hoxd11 expression**

(A) Real time quantitatve PCR revealed that *Hoxd11* mRNA was expressed in *Eed-cKO* cortical samples but not in CTRL samples. Values were normalised to housekeeper gene *Gadph*, and are presented as ΔCT (B-E) Immunofluorescent staining of DAPI (A, B) and Hoxd11 (C,D) in the somatosensory cortex of CTRL (A, C) and *Eed-cKO* mice. Hoxd11 staining was clearly observed in the *Eed-cKO* cortex but only sparsely in the CTRL cortex. (A) n = 2 CTRL, 2 *Eed-cKO* (B-E); n = 3 CTRL, 3 *Eed-cKO* (E).


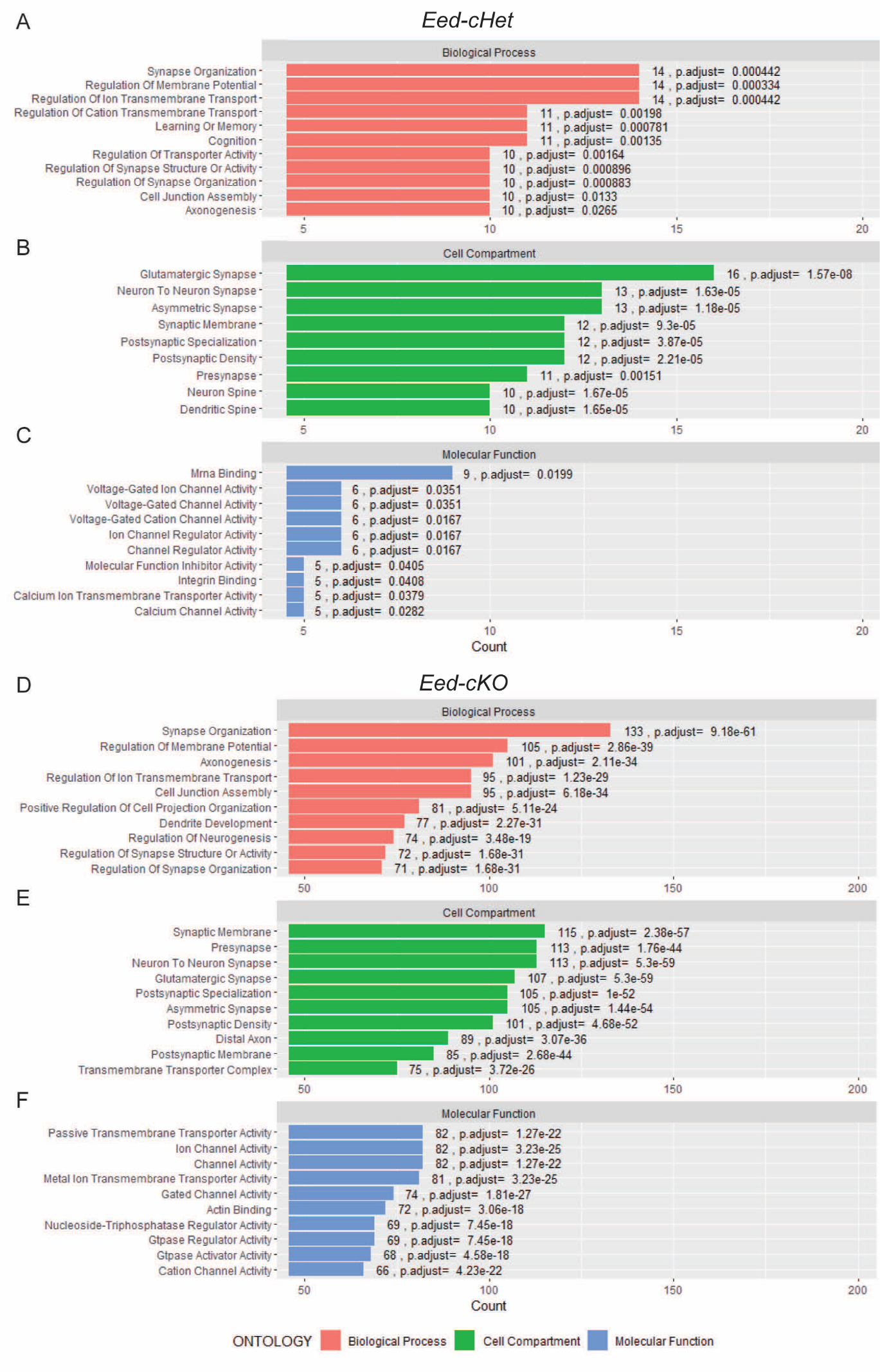


**Supplementary Figure 6 – Gene ontology terms enriched in *Eed-cKO* mice**

Top 10 enriched GO terms for *Eed-cHet* (A-C) and *Eed-cKO* (D-F) glutamatergic neurons. GO terms are categorized into biological processes (A, D), cell compartment (B, E), and molecular function (C, F). ‘Count’ refers to number of dysregulated genes that are associated with the GO term. Adjusted p-value is displayed next to each bar.


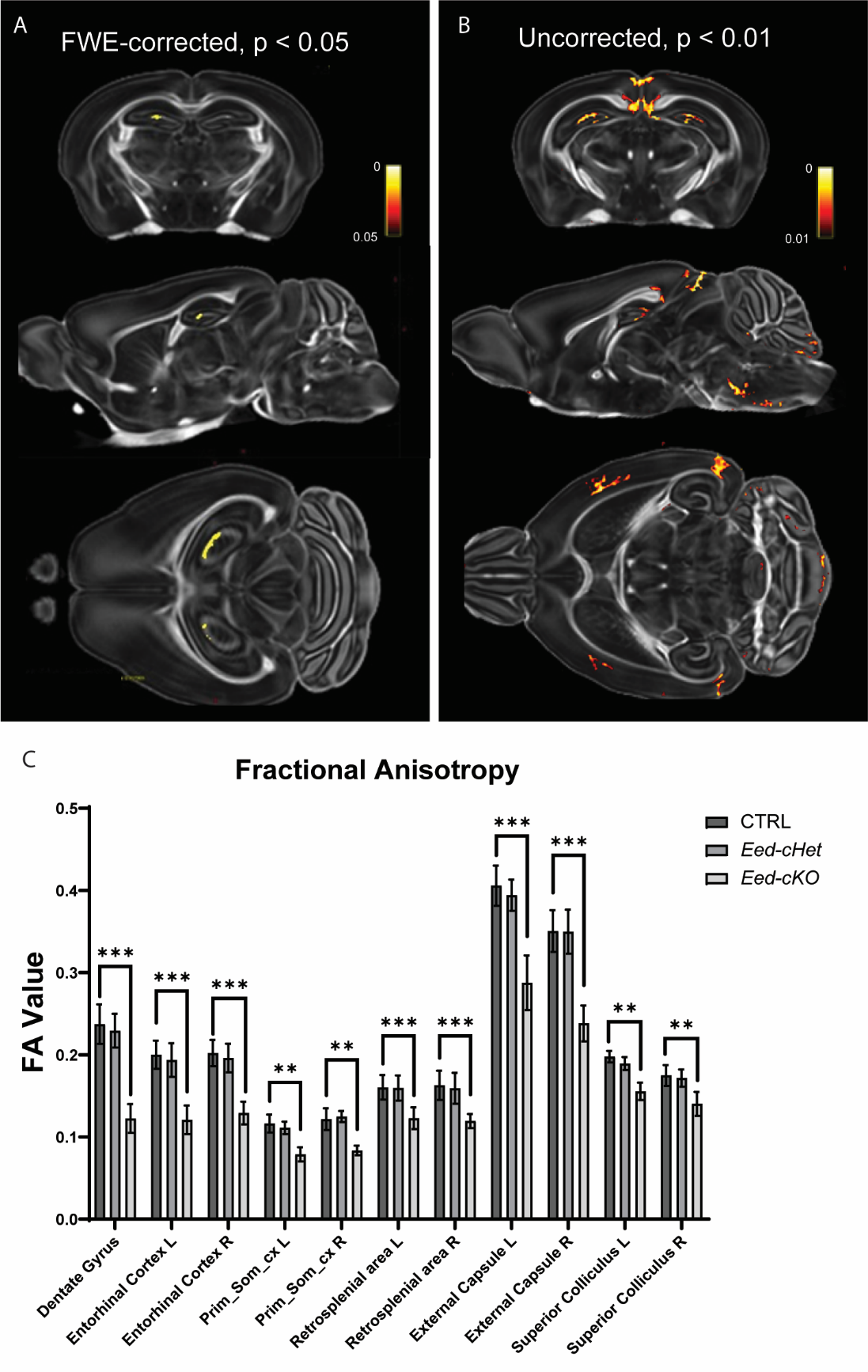


**Supplementary Figure 7 – Voxel-based morphometry analysis of fractional anisotropy**

(A) Map of significant changes in FA of *Eed-cKO* brains compared to control using a statistically stringent test corrected for family wise error (FWE) and p-value < 0.05. *Eed-cKO* mice had statistically reduced FA in the dentate gyrus. (B) Map of significant changes in FA of *Eed-cKO* brains compared to control using a less statistically stringent test, not corrected for FWE but with p-value < 0.01. (C) Graph showing each region where FA was statistically difference in *Eed-cKO* mice using the uncorrected test in (B). “L” and “R” refer to “left” and “right” respectively. “Prim_Som_cx” refers to the primary somatosensory cortex. Asterisks (*) indicate that p < 0.005. n = 7 CTRL, 8 *Eed-cHet*, 5 *Eed-cKO*.


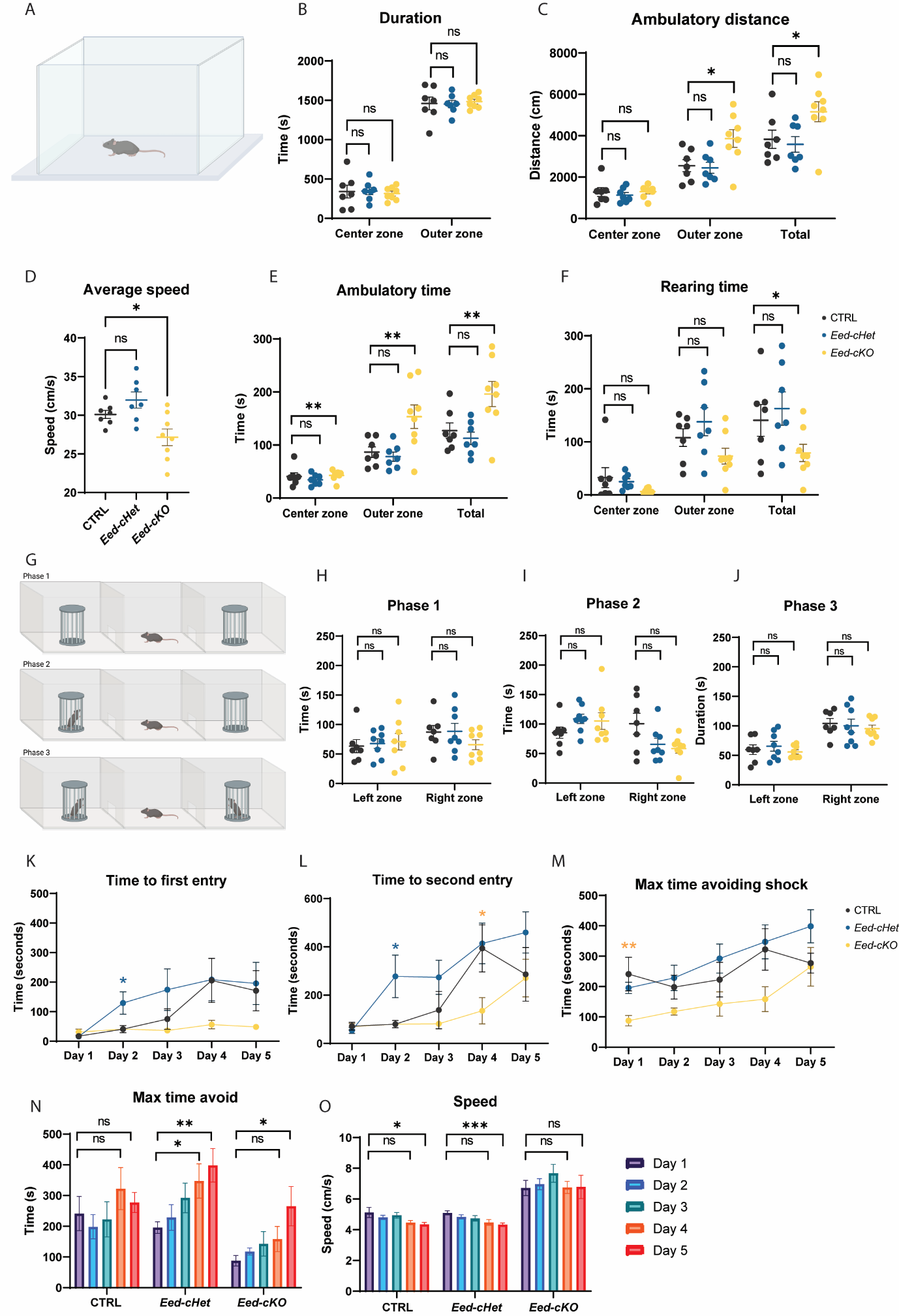


**Supplementary Figure 8 – Open field maze, 3-chamber social interaction test, and additional data for the active place avoidance test**

(A-F) Open field maze test. (A) Schematic of arena set up, which consists of an enclosed square arena. Duration in outer and inner zone (B), distance moved (C), and average speed (D) was measured. (E-F) Time spent performing activities including ambulatory time (E) and rearing time (F) was also measured. There was no significant difference in time spent on stereotypic movements, jumping or resting between genotypes (data not shown). (G-J) 3-chamber social interaction test. (G) Schematic of arena layout, which consists of 3 compartments, with cages in the outer compartments. The test consists of three phases in which the test mouse was acclimatised to the arena (phase 1), before being exposed to a novel mouse in the left cage (phase 2), and then a second novel mouse in the right cage (phase 3). Time spent next to the left and right cages (“left zone” and “right zone”, respectively) was measured. (K-O) Additional data for the active place avoidance test, as presented in Figure 7G-L. Intergenotype comparisons for time to first entry (K), time to second entry (L), and max time avoiding shock (M) are shown. Intragenotype comparisons for max time avoiding shock (N), and average speed (O) are shown. *** = p<0.001, * = p<0.05, ns = not significant, one-way ANOVA. n = 8 CTRL, 8 *Eed-cHet*, 8 *Eed-cKO*.


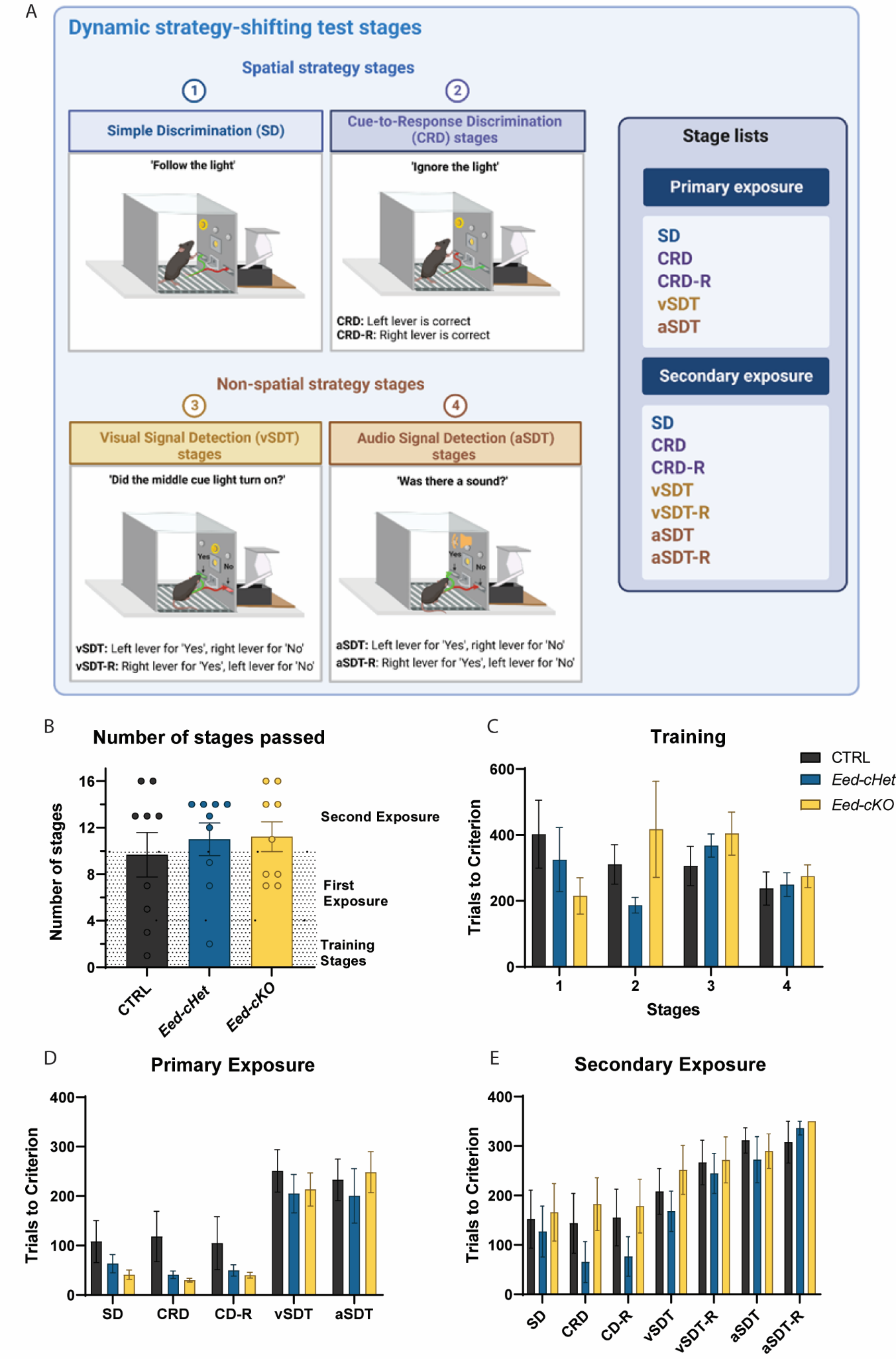


**Supplementary Figure 9 – Dynamic strategy shifting test**

(A) Schematic of arena and criteria for the primary and secondary exposure phases. (B) Number of stages passed. All three genotypes were able to complete all phases (training stage, first exposure, and second exposure). (C-E) Number of trials to criterion was measured for the training (C), primary exposure (D), or secondary exposure (E) phases. Statistical analysis was conducted with Brown-Forsythe ANOVA (B) or Friedman tests and Wilcoxon signed rank tests with Dunn’s multiple comparisons test where appropriate, as windsorisation resulted in non-gaussian distributions (C-E). n= 9 CTRL, 9 *Eed-cHet*, 9 *Eed-cKO* (A); n= 7 CTRL, 8 *Eed-cHet*, 9 *Eed-cKO* (C-E).

# Supplementary Tables

**Supplementary Table 1: Expression of Prox1, Calbindin, and Ctip2 in the hippocampal regions of *Eed-cKO* mice.**

|  | **Prox1** | **Calbindin** | **Ctip2 (CA1/2 region)** |
| --- | --- | --- | --- |
| **Total examined** | 10 | 8 | 6 |
| **Expression in both hemispheres** | 3 | 3 | 4 |
| **Expression in one hemisphere** | 4 | 4 | 2 |
| **No expression** | 3 | 1 | 0 |

**Supplementary Table 2: Top 10 DEGs of each glutamatergic neuron cluster.**

| **L2/3** | **L4/5** | **L6** |
| --- | --- | --- |
| *Gpc6*  *Chrm3*  *Lrrtm4*  *Dgkb*  *Rgs6*  *Sorcs3*  *Pam*  *Slit3*  *Gria3*  *Ntrk3* | *Rorb*  *Zmat4*  *Nell1*  *Cdh12*  *Cacna2d3*  *Astn2*  *Hs6st3*  *Brinp3*  *Rcan2*  *Kcnh5* | *Il1rapl2*  *Zfp804b*  *Galnt14*  *Sorcs3*  *Dpp10*  *Ptprm*  *Pigk*  *Slc24a2*  *Hs3st2*  *Sema3e* |
| **L6** | **Hom #1** | **Hom #2** |
| *Zfpm2*  *Hs3st4*  *Cdh18*  *Thsd7b*  *Mgat4c*  *Tle4*  *Foxp2*  *Frmpd4*  *Dlc1*  *Dpp10* | *Dach1*  *Nkain3*  *Galntl6*  *Zbtb20*  *Grid2*  *Nnat*  *Rmst*  *Kcnmb2*  *Adarb2*  *Tenm3* | *Ntng1*  *Cntn5*  *Inpp4b*  *Tafa2*  *Unc13c*  *Erbb4*  *Zfp536*  *Ndst4*  *Il1rapl2*  *Thsd7a* |

# Supplementary Methods

## Immunofluorescent staining

**Supplementary Table 3: List of antibodies used in immunofluorescent staining.**

|  | Dilution | Source | Identifier |
| --- | --- | --- | --- |
| Primary antibodies |  |  |  |
| Rabbit anti-H3k27me3 | 1/300 | Millipore | Cat#: 07-449 |
| Rat anti-Ctip2 | 1/300 | Abcam | Cat#: ab18465 |
| Rabbit anti-Satb2 | 1/300 | Abcam | Cat#: ab34735 |
| Rabbit anti-Foxp2 | 1/300 | ThermoFisher Scientific | Cat#: MA5-31419 |
| Mouse anti-Parvalbumin | 1/300 | Sigma-Aldrich | Cat# P3088 |
| Mouse anti-Calbindin | 1/300 | Sigma-Aldrich | Cat#: 9848 |
| Mouse anti-Gfap | 1/300 | Cell signalling | Cat#: 36705 |
| Rabbit anti-Olig2 | 1/300 | Merck Millipore | Cat#: ab9610 |
| Rabbit anti-Prox1 | 1/300 | Abcam | Cat#: ab199359 |
| Rabbit anti-Hoxd11 | 1/500 | ThermoFisher Scientific | Cat#: PA5-144013 |
| Secondary antibodies |  |  |  |
| Donkey anti-Rabbit 647 | 1/400 | Abcam | Cat#: ab150075 |
| Donkey anti-Rabbit 555 | 1/400 | Abcam | Cat#: ab150074 |
| Donkey anti-Rabit 488 | 1/400 | Abcam | Cat#: ab150073 |
| Donkey anti-Mouse 647 | 1/400 | Abcam | Cat#: ab150107 |
| Donkey anti-Mouse 488 | 1/400 | Abcam | Cat#: ab150105 |
| Donkey anti-Rat 555 | 1/400 | Abcam | Cat#: ab150154 |
| Donkey anti-Goat 594 | 1/400 | Abcam | Cat#: ab150132 |
| DAPI | 0.1-1 µg/mL | Sigma-Aldrich | Cat#: D9542 |

## Golgi Cox staining

Golgi Cox solution was prepared by combining 5% Potassium dichromate, 5% Mercuric chloride, and 5% Potassium chromate. This solution was stored in the dark for 5 days, then filtered, to create the working solution. Perfusions were performed with 0.04% PFA. Freshly perfused brains were incubated in Golgi-Cox solution for 10 days at room temperature, then incubated in 30% sucrose for 4 days at 4°C. Brains were vibratome sectioned (150 µm) and mounted onto SuperFrost Plus slides immersed in 1.25% gelatin and 0.1% chromium. Mounted tissue was dried for 2 days at 4°C in the dark. Sections were rehydrated, then placed in 0.1 M ammonia solution for 30 minutes. Sections were rinsed, then placed in Fujifilm Automatic X-Ray fixer solution for 30 minutes. Sections were rinsed again, then dehydrated through an ethanol series, washed in xylene, and then mounted with dibutylphthalate polystyrene xylene. Imaging was performed with a Nikon Upright Stereology microscope. Pyramidal neurons from layer 2/3 and layer 5 were imaged at 40X magnification with 2 μm step Z-stacks. Additionally, basal dendrites were imaged with 100x magnification with 0.3 μm Z-stacks. In both cases, the number of Z-stacks depended on how many were required to image the whole neuron/dendrite. Tracing and analysis was performed with Neurolucida 360 software (version 2019) (Dickstein et al. 2016). To analyse orientation, the motor cortex of each brain was imaged at 10X magnification with 5 μm step Z-stacks. 0.1 mm^2^ areas within layers 2/3 and 5 of the motor cortex were randomly selected, and the orientation of pyramidal neurons within was quantified as described (Ye et al. 2008). A line was drawn extending out from the centre of the cell soma that was perpendicular to the outer surface of the cortex. Another line was drawn from the centre of the soma along the direction of the apical dendrite. The angle between these two lines was then measured.

## DTMRI

### Tissue preparation

Mice were perfused as described above; however the skull was left intact. Samples were post-fixed in 4% PFA for 24 hours, and then stored in 0.1 M PBS with 0.02% sodium azide. Before scanning, skulls were immersed in 0.1 M PBS with 0.2% gadobutrol (Gadovist, Bayer, Ontario, Canada) for 8 weeks to enhance MRI contrast.

### Diffusion MRI Acquisition

Brain data was acquired using a Bruker Ultrashield Plus 700 WB Avance NMR spectrometer (16.4 T vertical bore animal microimaging system). It was equipped with a 15 mm linear surface acoustic wave coil, a micro2.5 gradient insert that provides a 2.5 mT/m/A gradient strength, and a 60 A gradient amplifier. Two sets of scans were obtained: a high-resolution 3D T1 weighted Fast Low Angle Shot (FLASH) for structural information imaging; and 3D Stejskal-Tanner diffusion weighted imaging (DWI) spin-echo in multiple directions for fibre tracking purposes. Samples were scanned using a high-resolution FLASH T1 weighted sequence and a DWI protocol as illustrated in Supplementary Table 4 and Supplementary Table 5 below.

**Supplementary Table 4: High resolution T1 imaging sequence parameters.**

| High resolution T1 | | | | | |
| --- | --- | --- | --- | --- | --- |
| TR | TE | Matrix Size | FOV | Resolution | Bandwidth |
| 50 ms | 12 ms | 654×433×333 | 19.6×13×10 mm | 30 μm Isotropic | 50 KHz |

**Supplementary Table 5: DWI sequence parameters.**

| DWI | | | | | | |
| --- | --- | --- | --- | --- | --- | --- |
| TR | TE | *b* value | Matrix Size | FOV | Resolution | Bandwidth |
| 200 ms | 21 ms | 5000 s/mm^2^  3xb_0_ 30 directions | 196×130×100 | 19.6×13×10 mm | 100 μm | 50 KHz |

### Data pre-processing and analysis

DWI data were zero-filled by a factor of 1.5 prior to Fourier transform. Non-brain tissue was removed through masking brain tissue only using ITKsnap software package (Yushkevich et al. 2006) then the diffusion data were denoised and bias-field corrected using both MRtrix and ANT’s software packages (Avants et al. 2014; Tournier et al. 2019). MRtrix was used to calculate the response function as well as the iFOD2 map (Tournier et al. 2019).

A 106 regions of interest (ROIs) parcellation image, created from an MRI based atlas of adult C57BL/6 mouse (Liu et al. 2016), was used for probabilistic tractography. Both the atlas and the parcellation images were registered to each subject’s b_0_ image using FMRIB Software Library (FSL) Linear Image Registration Tools (FLIRT) rigid-body registration, followed by affine and nonlinear deformation using ANTs (Avants and Tustison ; Jenkinson et al. 2002; Jenkinson and Smith 2001). The ROIs were defined as nodes between which the edges were computed.

### Structural connectome and VBM analysis

The connectome was studied network-wise by graph theory analysis and connection-wise using the Network-Based Statistic Toolbox (NBS) (Wang et al. 2015; Zalesky et al. 2010). For NBS, connectomes were compared over multi-thresholds ranging between t = 2.1 to t = 6.5 with an increment of 0.1 each time to compute the FWER-corrected p-value for each component. For graph theory analysis, network metrics were measured over 10 thresholds ranging between t = 0.05 to t = 0.5 of the network sparsity. These metrics include degree centrality, betweenness, small worldness, and modularity.

### Volumetric analysis

The volumetric change in *Eed-cHet* and *Eed-cKO* mice compared to CTRL mice was also investigated in 20 regions using a comprehensive three-dimensional digital atlas by Ma et al (Ma et al. 2005).

### DTMRI Metrics

DTMRI maps were calculated using MRTrix3, and DTMRI metrics were measured using the FSL software. DTMRI metrics were measured from the corpus callosum, hippocampal commissure, and anterior commissure. Fixel-based analysis was performed using MRTrix3 to investigate WM fibre density and morphology (Raffelt et al. 2017). FOD maps were used to create an unbiased FOD 4D template then each subject’s FOD map was registered to the template and warped to the template’s space. These warped-FOD images were then segmented to estimate fixels and their AFD while “warp” images were used to compute the fibre cross-section. Probabilistic Tractography was performed on the FOD template to generate a whole-brain tractogram. This tractogram was used to generate a fixel-fixel connectivity matrix. Finally, fixel data was smoothed then statistical analysis was performed using connectivity based fixel enhancement for each measure (AFD and cross-section) (Raffelt et al. 2017).

Voxel-based morphometry (VBM) analysis was performed using Threshold-Free Cluster Enhancement (TFCE) with controlled family-wise error (FWE) rate to detect structures of the brain that were most significantly affected by *Eed* ablation (Winkler et al. 2014). Multivariate templates were created using ANTs (Avants and Tustison ; Jenkinson et al. 2002; Jenkinson and Smith 2001). DTMRI-derived maps were registered together in a non-biased space firstly using linear transformation (rigid and affine) followed by a deformable transformation with higher degrees of freedom (Avants et al. 2011). FSL randomise was then performed to calculate the FWE-TFCE test statistic maps and a group of p value maps (Winkler et al. 2014).

## Single nuclei-RNA-Seq

### Single nuclei extraction

Tissue was dissected and immediately transferred into the Dounce homogenizer, filled with 1 ml of homogenisation buffer [250 mM sucrose, 25 mM KCl, 5 mM MgCl2, 10 mM Tris buffer (pH 8), 1 µM DTT, 1x protease inhibitor (cOmplete Protease Inhibitor Cocktail Tablets, Roche), 0.1% (v/v) Triton X-100 10%, and 40 U/mL RNase (RNasIn, Promega), diluted in nuclease-free water]. The tissue was homogenised with 10 strokes of the loose pestle, followed by 15 strokes of the tight pestle. The homogenate was transferred to a prechilled 15 ml tube and 4 ml of cold homogenisation buffer was added, then incubated for 5 minutes on ice. The homogenate was then strained through a 40 µm cell strainer prewetted with 0.5 ml homogenisation buffer, into a 50 ml tube. The strainer was washed with 0.5 ml homogenisation buffer. The homogenate was centrifuged for 5 minutes at 500 g in a swing bucket. The cell pellet was resuspended in 2ml wash media [1% BSA and 100 U/mL RNasIn, diluted in PBS], and DAPI was added at 1:5000 concentration in 1 ml wash media. The cell suspension was submitted for FACS sorting, using DAPI to filter nuclei from debris and to avoid collection of doublet nuclei. Following this, the samples were submitted to the Institute for Molecular Bioscience Sequencing Facility for cDNA library preparation with the 10x Genomics Chromium platform using a 3’ v3.1 kit. Approximately 7000 nuclei per sample were loaded to achieve a target barcoded library of approximately 3500 nuclei per sample. The libraries were then sequenced on a NovaSeq 6000 with a sequencing depth of approximately 78,000-100,000 reads/nuclei.

### Analysis of snRNA-seq data

Analysis was performed with the Seurat toolkit for single cell genomics on R Studio (Hao et al. 2021). Firstly, CTRL, *Eed-cHet*, and *Eed-cKO* data was merged into one Seurat object. Cells with <2% mitochondrial RNA were filtered out as these were likely to be low-quality cells such as dead or dying cells. The data was normalised with both SCTransform (Hafemeister and Satija 2019) (for cluster calculations) and NormaliseData (for data visualisation). Cells were then clustered with the standard Seurat workflow (RunPCA, RunUMAP, FindNeighbors, FindClusters) (dims = 1:30, resolution = 0.2). Clusters were identified based on canonical gene expression, and by using Linnarsson lab scRNA-seq data of the mouse cortex and hippocampus for reference of gene expression in different cell types (Zeisel et al. 2015). Clusters with unusual nFeature, nCount, or abnormal gene expression were filtered out as these were likely to be doublets or abnormal cells. Differentially expressed gene (DEG) analysis performed with FindMarkers (wilcox test, logfc.threshold = 0.2). Then, glutamatergic neuron clusters were isolated by subsetting them and forming new Seurat object. The workflow for analysing glutamatergic neurons was similar to that described above. Azimuth (Hao et al. 2021) was used to transfer annotations from CTRL to *Eed-cKO* glutamatergic neurons. Finally, Gene ontology (GO) analysis was performed with the clusterProlifer function enrichGO (Wu et al. 2021).

## Behaviour

### SHIRPA

Mice were screened for gross neurological deficits using SHIRPA (Smith Kline Beecham Pharmaceuticals; Harwell, MRC Mouse Genome Centre and Mammalian Genetics Unit Imperial College School of Medicine at St. Mary’s; Royal London Hospital, St. Bartholomew’s and the Royal London School of Medicine phenotype assessment) screen (Rogers et al. 1997). Mice underwent a series of tests and observations to assess their health, sensory and motor function, neurological reflexes, and autonomic functions (Rogers et al. 1997). No neurological deficits were found in CTRL, *Eed-cHet*, or *Eed-cKO* mice.

### Open Field Test

The open field test (Seibenhener and Wooten 2015) consists of an enclosed arena in which mouse movements were tracked with infrared sensors. Factors such as ambulatory movement (walking or running), stereotypic movement (small movements such as scratching), rearing, jumping, and resting were recorded. Additionally, the location of the mouse in the arena was recorded. Each mouse was recorded in the activity monitor arena for 30 minutes. The lighting at the level of the arena was 80 lux.

### Light/Dark Box

The light/dark box (Bourin and Hascoët 2003) consists of two compartments separated by a small door. The ‘light’ compartment was brightly lit with an open roof, and the ‘dark’ compartment was covered and dark. Like the activity monitor, mouse activity was recorded with infrared sensors. Ambulatory, stereotypic, resting, rearing, and jumping movements were recorded. This test was performed for 10 minutes. The lighting in the light compartment of the arena was 80 lux.

### Elevated Plus Maze

The elevated plus maze (Walf and Frye 2007) arena is in the shape of a plus symbol (+), with two open and two enclosed arms. The arena is placed on a pedestal 1 meter above the ground. Normally mice are averse to open spaces and prefer the enclosed arms. Hence, this test investigates anxious vs exploratory behaviours. This test was performed for 10 minutes per mouse. The lighting of the arena was 80 lux.

### 3-Chambered Social Interaction Test

The three-chambered apparatus consisted of three chambers, each compartment was 20x30x30 cm, with a wire frame steel cage in the left and right chambers (Kaidanovich-Beilin et al. 2011). Firstly, the subject mouse was placed in the centre of the apparatus and allowed to habituate for 5 minutes. After 5 minutes, the subject mouse was confined to the centre chamber using dividers. A conspecific mouse of the same sex was placed in either the left or right chamber within a wire framed steel cage. The dividers were removed, and the subject mouse was allowed to move freely between the three chambers for 5 minutes. Next the subject mouse was confined to the central chamber again, and a second conspecific mouse of the same sex was placed in the remaining wired framed steel cage. The dividers were removed, and the subject mouse was able to move freely between the three chambers for a further 5 minutes. Video recordings were coded and analysed blind to treatment using Ethovision XT (Noldus Information Technology, NLD) to track the centre of mass (body). The cumulative duration and frequency of the mouse to enter each zone was measured as well as the amount of time spent near the conspecific mouse. The lighting at the level of the three-chambered apparatus was 100 lux.

### Active Place Avoidance (APA)

The APA task was employed to assess hippocampal-dependent spatial learning (Stuchlík et al. 2013; Wesierska et al. 2005). The APA apparatus consists of an elevated spherical arena (77 cm in diameter and 32 cm in height) with a grid floor. The arena rotated clockwise at 1 rpm. The task of the mouse was to avoid an invisible shock zone by using four external cues which were hung on nearby walls of the room. If the mouse entered the shock zone, it would receive a mild foot shock (500 ms, 60 Hz, 0.5 mA) via the grid floor. The mouse received a further shock if it did not escape the shock zone [same intensity with 1.5-second intervals]. An overhead camera was used to track the mouse and a computer-based software was used to analyse the image and to deliver the mild shock appropriately (Tracker, Bio-Signal Group Corp., Brooklyn, NY). The experiment was conducted over 6 consecutive days, habituation (Day 0, without shock for 5) and training (Day-1 to 5, with shock 10 minutes/day). Behavioural parameters such as “number of shocks received”, “latency to enter into the shock zone”, “max time avoid”, and “distance travelled” were analysed at the end of the experiment using software (Track Analysis) provided by the Bio-Signal Group. The lighting at the level of the arena was 60 lux.

### Cognitive Flexibility Test (Dynamic Strategy-Shifting Task)

#### Housing

Prior to, and during operant training, mice were food deprived and weights were reduced and maintained at 90% of their free-feeding weights to enhance motivational salience of operant rewards. Training and testing were conducted from Monday-Saturday 1-2 times daily for the duration of experiments.

#### Apparatus

Behavioural testing was conducted in modular operant chambers (Med Associates Inc., St. Albans, VT, USA). The Dynamic Strategy-Shifting Task (DSST) was conducted within six chambers (24.1 cm x 20.3 x 18.4 cm). Strawberry milk (Breaka, Queensland, Australia) was delivered as positive reinforcement via a motorised dipper mechanism by raising the dipper cup (~0.06 cc) into the reward magazine. Reward magazines, nose-poke apertures and levers were all fitted with infrared head-entry detectors to record chamber interactions. The chambers also featured white-noise generators which were fitted externally to the back wall for the audio aspect of the task. All chambers were enclosed within sound-attenuating boxes (55.9 cm x 55.9 cm x 35.6 cm), each fitted with a fan to provide ventilation and buffering from external noise, and an infrared camera (D-link Australia, North Ryde, NSW, Australia) for recording and observation. Protocols were written on MED-PC (Med Associates Inc. VT; S. Alexander) with a data collection resolution of 0.01 s.

#### Dynamic Strategy-Shifting Task (DSST) test

Instrumental training began with a “reward training” stage where the mice learned to collect rewards by making head entries (HEs) into the reward magazine to trigger the dipper mechanism. Mice were progressed to “self-initiation training” after completing the reward training stage. Here, they would learn how to ‘self-initiate a trial by making a HE into the centre nose-poke before making a HE into the magazine to collect a reward. The third and fourth stimulus-response training stages required the mice to first self-initiate a trial for the levers to be extended. Mice were then required to press either lever beside the magazine before making a HE into the magazine to collect a reward. The fourth stage differed only from the third stage by the introduction of further restrictions on the time to press a lever (limited hold) and to collect a reward. After completing these four stages, the mice would progress to the testing phase of the Dynamic strategy-shifting task.

The test phase of the DSST was delivered in two phases referred to as the primary and secondary exposures consisting of 5 and 7 stages, respectively. Completion of the primary exposure was a requirement to progress to the secondary exposure. During primary exposure, mice were subjected to all spatial and non-spatial discrimination stages but not to non-spatial reversal stages until the secondary exposure. Criterion to pass each stage was 6 consecutive correct responses. If a mouse passed a stage within 30 minutes, it would progress to the next stage within that session. A session end was signalled if the mouse either failed to pass a stage within 30 minutes, made over 120 trials within one stage, had been inactive for over 5 minutes or the exceeded the maximum session time (90 minutes). Prior to commencement of test, mice were split into two groups with different starting target locations to control for any potential side bias. Number of trials to criteria (TTC) for each stage was the key behavioural outcome extracted from both training and testing phases.

#### Statistical Analyses

Statistical analyses was conducted using the SPSS software package (ver.28, SPSS Inc. IL,USA) and GraphPad Prism (ver.9.3.1) with significant set to *p*<0.05. Only mice that had completed at least half of the stages in each exposure were included in TTC comparisons. Mice were given a maximum TTC of 350 if they never completed the stage or if they exceeded that amount for the purpose of conducting comparisons and reducing the effects of extreme outliers. Comparisons of TTC were conducted using Friedman tests and Wilcoxon signed rank tests with Dunn’s multiple comparisons test where appropriate, as windsorisation resulted in non-gaussian distributions. Number of sessions was compared between genotypes using a Brown-Forsythe ANOVA.
